# Breathomics for Assessing the Effects of Treatment and Withdrawal With Inhaled Beclomethasone/Formoterol in Patients With COPD

**DOI:** 10.3389/fphar.2018.00258

**Published:** 2018-04-17

**Authors:** Paolo Montuschi, Giuseppe Santini, Nadia Mores, Alessia Vignoli, Francesco Macagno, Rugia Shoreh, Leonardo Tenori, Gina Zini, Leonello Fuso, Chiara Mondino, Corrado Di Natale, Arnaldo D'Amico, Claudio Luchinat, Peter J. Barnes, Tim Higenbottam

**Affiliations:** ^1^Department of Pharmacology, Faculty of Medicine, Catholic University of the Sacred Heart, University Hospital Agostino Gemelli Foundation, Rome, Italy; ^2^Magnetic Resonance Center (CERM), University of Florence, Florence, Italy; ^3^Department of Internal Medicine and Geriatrics, Catholic University of the Sacred Heart, University Hospital Agostino Gemelli Foundation, Rome, Italy; ^4^Department of Drug Sciences, Faculty of Pharmacy, University “G. d'Annunzio”, Chieti, Italy; ^5^Department of Hematology, Faculty of Medicine, Catholic University of the Sacred Heart, University Hospital Agostino Gemelli Foundation, Rome, Italy; ^6^Department of Allergology, ‘Bellinzona e Valli’ Hospital, Bellinzona, Switzerland; ^7^Department of Electronic Engineering, University of Tor Vergata, Rome, Italy; ^8^Airway Disease Section, Faculty of Medicine, National Heart and Lung Institute, Imperial College, London, United Kingdom; ^9^Faculty of Pharmaceutical Medicine, Royal College of Physicians, London, United Kingdom

**Keywords:** breathomics, inhaled corticosteroids, long-acting β_2_-agonists, COPD, pharmacotherapy

## Abstract

**Background:** Prospective pharmacological studies on breathomics profiles in COPD patients have not been previously reported. We assessed the effects of treatment and withdrawal of an extrafine inhaled corticosteroid (ICS)-long-acting β_2_-agonist (LABA) fixed dose combination (FDC) using a multidimensional classification model including breathomics.

**Methods:** A pilot, proof-of-concept, pharmacological study was undertaken in 14 COPD patients on maintenance treatment with inhaled fluticasone propionate/salmeterol (500/50 μg b.i.d.) for at least 8 weeks (visit 1). Patients received 2-week treatment with inhaled beclomethasone dipropionate/formoterol (100/6 μg b.i.d.) (visit 2), 4-week treatment with formoterol alone (6 μg b.i.d.) (visit 3), and 4-week treatment with beclomethasone/formoterol (100/6 μg b.i.d.) (visit 4). Exhaled breath analysis with two e-noses, based on different technologies, and exhaled breath condensate (EBC) NMR-based metabolomics were performed. Sputum cell counts, sputum supernatant and EBC prostaglandin E_2_ (PGE_2_) and 15-F_2t_-isoprostane, fraction of exhaled nitric oxide, and spirometry were measured.

**Results:** Compared with formoterol alone, EBC acetate and sputum PGE_2_, reflecting airway inflammation, were reduced after 4-week beclomethasone/formoterol. Three independent breathomics techniques showed that extrafine beclomethasone/formoterol short-term treatment was associated with different breathprints compared with regular fluticasone propionate/salmeterol. Either ICS/LABA FDC vs. formoterol alone was associated with increased pre-bronchodilator FEF_25−75%_ and FEV_1_/FVC (*P* = 0.008–0.029). The multidimensional model distinguished fluticasone propionate/salmeterol vs. beclomethasone/formoterol, fluticasone propionate/salmeterol vs. formoterol, and formoterol vs. beclomethasone/formoterol (accuracy > 70%, *P* < 0.01).

**Conclusions:** Breathomics could be used for assessing ICS treatment and withdrawal in COPD patients. Large, controlled, prospective pharmacological trials are required to clarify the biological implications of breathomics changes. EUDRACT number: 2012-001749-42.

## Introduction

Respiratory inflammation has a central role in the pathophysiology of COPD. Inhaled corticosteroids (ICS) are the principal anti-inflammatory treatment for COPD (Global initiative for chronic obstructive lung disease (GOLD), 2018), but little is known about the pattern of inflammatory markers which are affected by this pharmacotherapy. While ICS are generally effective in asthma, their anti-inflammatory efficacy in COPD is not clearly established (Barnes, 2010).

Electronic noses (e-noses), consisting of cross-reactive chemical sensor arrays for the detection of volatile chemical species (Horvath et al., 2017), discriminate between patients with COPD, asthmatic patients, healthy smokers and healthy non-smokers (Fens et al., 2009), and predict oral corticosteroid responsiveness in asthmatic patients (van der Schee et al., 2013). E-nose breathprints are associated with airway inflammatory markers, including sputum eosinophils and neutrophils, in COPD patients and can be useful for subphenotyping (Fens et al., 2011, 2013). Metabolomics is a global approach to understanding regulation of metabolic pathways and metabolic networks of a biological system (Rochfort, 2005). Nuclear magnetic resonance (NMR)-based metabolomic analysis of exhaled breath condensate (EBC), the liquid fraction of exhaled breath, is suitable for identifying and quantifying semi-volatile/non-volatile small molecular weight metabolites and has been applied to the assessment of airway inflammation in COPD patients (De Laurentiis et al., 2008; Motta et al., 2012; Airoldi et al., 2016). Published prospective clinical studies on effects of pharmacological treatment on breathomics in COPD patients are not available.

We aimed to assess the effects of treatment and steroid withdrawal of an extrafine ICS/long-acting β_2_-agonist (LABA) fixed dose combination (FDC) on breathprints and non-invasive inflammatory outcomes in COPD patients; to compare accuracy of a multidimensional classification model vs. a standard spirometry-based model.

## Methods

### Study subjects

Fourteen COPD ex-smokers for at least 1 year with stable mild, moderate or severe airflow limitation (GOLD stage I-III, post-bronchodilator forced expiratory volume in 1 s (FEV_1_) >30% predicted value, FEV_1_/FVC < 70%, GOLD group A-D), who were on a fixed dose combination (FDC) of fluticasone propionate/salmeterol delivered via a dry powder inhaler (DPI) at a constant dose of 500/50 μg b.i.d. for at least 8 weeks, were studied. Diagnosis of COPD was based on GOLD guideline criteria (Global initiative for chronic obstructive lung disease (GOLD), 2018). Six subjects had clinical and radiological signs of emphysema. Three patients had bronchiectasis documented by chest CT scan. Regarding co-morbidities, six patients with COPD had concomitant ischemic heart disease, four patients had congestive heart failure, five patients had arterial blood hypertension, three patients had type 2 diabetes mellitus, and one patient had peripheral vascular disease.

COPD patients had negative reversibility test to 400 μg of inhaled salbutamol (< 12% and 200 ml increase in FEV_1_), no history of asthma and atopic disease, negative skin prick test results, no upper respiratory tract infections in the previous 3 weeks, and were excluded from the study if they had used systemic corticosteroids in the previous 4 weeks.

### Study design

**Figure 1:**
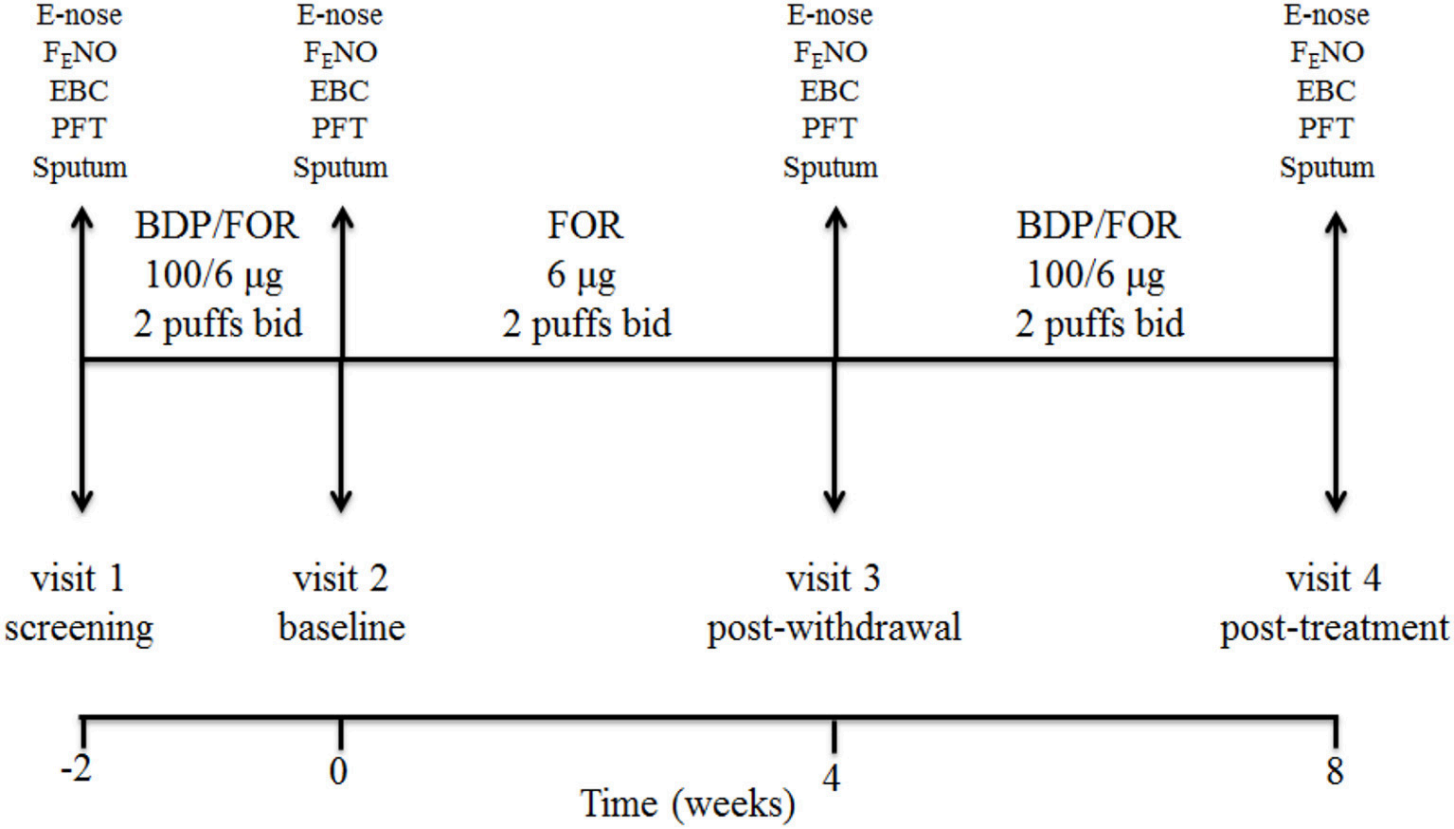
Study design and detail of interventions. At screening visit (visit 1), treatment with a constant dose of inhaled fluticasone propionate/salmeterol xinafoate fixed dose combination (FDC) (500/50 μg b.i.d. via a dry powder inhaler) for at least 8 weeks was switched to an extrafine formulation of inhaled beclomethasone dipropionate (BDP)/formoterol fumarate (FOR) FDC (100/6 μg 2 puffs b.i.d. via a pressurized metered dose inhaler [pMDI]). After a run-in phase of 2 weeks, the FDC containing beclomethasone was suspended (visit 2, baseline visit), while maintaining formoterol alone (6 μg 2 puffs b.i.d. via pMDI). After 4 weeks of beclomethasone withdrawal (visit 3, post-withdrawal visit), treatment with inhaled beclomethasone/formoterol FDC at the same dose (100/6 μg 2 puffs b.i.d. via pMDI) was reintroduced. Exhaled breath sampling for e-nose analysis, measurement of fraction of exhaled nitric oxide (F_E_NO), exhaled breath condensate (EBC) collection, pulmonary function tests (PFT), and sputum induction were performed at each visit. Study duration was 10 weeks.

This was a single center, prospective, open label, pilot, proof-of-concept, pharmacological study of ICS withdrawal and treatment in COPD patients on maintenance treatment with inhaled fluticasone propionate/salmeterol at full doses (visit 1, screening visit). Study duration was 10 weeks including 4 visits (Figure 1). After a 2-week phase treatment with inhaled beclomethasone/formoterol (100/6 μg 2 puffs b.i.d. via pressurized metered dose inhaler [pMDI]) (visit 2, baseline visit) to allow them to become familiar with the new inhaler, patients received inhaled formoterol alone (6 μg 2 puffs b.i.d. via pMDI) for 4 weeks (visit 3, post-withdrawal visit), and inhaled beclomethasone/formoterol (100/6 μg 2 puffs b.i.d.) for 4 weeks (visit 4, post-treatment visit) (Figure 1). Breathomics included analysis of exhaled breath gaseous phase with two different e-noses, and NMR-based metabolomics of exhaled breath condensate (EBC). Sputum cell counts, prostaglandin (PG) E_2_ and 15-F_2t_-isoprostane in sputum supernatants and EBC, and fraction of exhaled nitric oxide (F_E_NO) were also measured.

Both informed and written consent was obtained from patients. The study was approved by the Ethics Committee (A.942/C.E./2012) of the Catholic University of the Sacred Heart, University Hospital Agostino Gemelli, Rome, Italy. EudraCT number: 2012-001749-42.

### Pulmonary function

Spirometry was performed with a Pony FX spirometer (Cosmed, Rome, Italy) and the best of three consecutive maneuvers chosen.

### F_E_NO measurement

F_E_NO was measured with the NIOX system (Aerocrine, Stockholm, Sweden) with a single breath on-line method at constant flow of 50 ml/sec according to American Thoracic Society guidelines (American Thoracic Society and European Respiratory Society, 2005; Dweik et al., 2011). Exhalations were repeated after 1 min relaxation period until the performance of three F_E_NO values varies less than 10% (American Thoracic Society and European Respiratory Society, 2005). F_E_NO measurements were obtained before spirometry.

### Collection of exhaled breath

Exhaled breath was collected from each subject at 8.30 a.m. as previously described (Fens et al., 2009; Bofan et al., 2013) and based on recent ERS technical standard (Horvath et al., 2017). No food or drinks were allowed at least 12 h prior to sampling.

Subjects were asked to breathe tidally volatile organic compound (VOC)-filtered air for 5 min, while wearing a nose-clip, into a 2-way non-rebreathing valve with an inspiratory VOC filter and an expiratory silica reservoir to reduce sample water vapor (Röck et al., 2008). Then, subjects were asked to inhale to maximal inspiration and perform a FVC maneuver into a Tedlar® bag. Two consecutive samples were collected 15 min apart and immediately analyzed.

### Electronic noses

The first sample was analyzed with a commercially available e-nose (Cyranose 320, Sensigent, Baldwin Park, USA) (Lewis, 2004; Fens et al., 2009) which consists of an array of 32 cross-reactive carbon black polymer composite sensors and detects resistance variations; the second sample was analyzed with an e-nose prototype (Ten 2011, University of Rome Tor Vergata, Italy) (Montuschi et al., 2010) which consists of an array of 8 cross-reactive quartz microbalance gas sensors coated by molecular films of metallo-porphyrins and detects frequency variations (Montuschi et al., 2010). Breathprints were analyzed by pattern recognition algorithms (Bishop, 2006).

Ambient VOCs were subtracted from measures and results were automatically adjusted for ambient VOCs.

### EBC sampling

Before EBC collection, subjects refrained from eating for at least 3 h. EBC was collected using a condenser (Ecoscreen, Jaeger, Hoechberg, Germany) (Motta et al., 2012), in a windowless clinic facility without disinfectant dispensers (Motta et al., 2012). Samples were snap frozen in liquid nitrogen to immediately “quench” metabolism and preserve the metabolite concentrations.

### Metabolomic analysis of EBC with NMR spectroscopy

^1^H-NMR spectra for EBC samples were acquired using a Bruker 600 MHz spectrometer operating at 600.13 MHz using standard nuclear Overhauser effect spectroscopy (NOESY) experiments and standard protocols for sample preparation (Bertini et al., 2014). Signals of interest were assigned on template one-dimensional NMR profiles and integrated to calculate their relative concentrations (Montuschi et al., 2012; Motta et al., 2012; Bertini et al., 2014). Multilevel partial least squares (PLS) analysis was used to analyse EBC spectra from the same subject obtained at paired visits.

### Measurement of PGE_2_ and 15-F_2t_-isoprostane in sputum supernatants and EBC

PGE_2_ and 15-F_2t_-isoprostane concentrations in sputum supernatants and EBC were measured with radioimmunoassays developed in our laboratory, previously validated and compared with gas chromatography/mass spectrometry and high performance liquid chromatography (Wang et al., 1995; Montuschi et al., 2003).

### Sputum cell analysis

Sputum induction, processing and analysis were performed according to the European Respiratory Society (ERS) guidelines (Djukanovic et al., 2002; Efthimiadis et al., 2002; Paggiaro et al., 2002).

Baseline FEV_1_ was recorded before sputum induction. Subjects were pre-treated with inhaled salbutamol (400 μg), and, after 10 min, a spirometry was repeated (Paggiaro et al., 2002). Subjects were asked to inhale hypertonic saline (3%) for 5 min and then, to rinse their mouths and try to expectorate into a sterilized box (Paggiaro et al., 2002). Five-minute inhalation sessions were repeated four times for a total of 20 min (Paggiaro et al., 2002). A spirometry was performed after each inhalation session to detect significant fall of FEV_1_. The procedure was stopped when approximately 1 g of plugs was collected, or if patients had symptoms or/and if FEV_1_ was reduced more than 20% over baseline values (Paggiaro et al., 2002). Sputum was processed within 2 h to ensure optimum cell counting and staining, with the sample always kept in ice (Efthimiadis et al., 2002). One hundred to 500 μg sputum was selected for sputum analysis. Dithiothreitol (DTT) 0.1% was added to sputum samples which were kept in a shaking rocker at room temperature for 20 min for sample homogenization (Efthimiadis et al., 2002). Samples were filtered through a 48 μm nylon mesh into a pre-weighed conical tube and filtrate was weighed. Total cell count was performed manually using a haemocytometer and cell viability was assessed by the trypan blue exclusion method before centrifugation (Efthimiadis et al., 2002). To separate cell pellet from sputum supernatants, samples were centrifuged at 4°C for 10 min with a centrifugal force of 1,200 × g (Efthimiadis et al., 2002). Sputum supernatant samples were collected and stored at −80°C for measurement of PGE_2_ and 15-F_2t_-isoprostane concentrations. Cell pellets were resuspended in PBS buffer and cell concentrations were adjusted to 1 × 10^6^ cells/ml. Cytospins were prepared by adding 40–60 μl of cell resuspension to each cytospin and using a Shandon cytocentrifuge at 22 × g for 6 min (Efthimiadis et al., 2002). Cytospins were stained for differential cell counts using Giemsa staining (Efthimiadis et al., 2002). The differential cell counts were performed by counting a minimum of 400 nonsquamous cells and reported as the relative numbers of eosinophils, neutrophils, macrophages, lymphocytes, and bronchial epithelial cells, expressed as a percentage of total non-squamous cells (Efthimiadis et al., 2002). The percentage of squamous cells was reported separately. Slides with squamous cells >30% of total cells were discarded. Slides were read blindly by two qualified and fully trained physicians. Monthly quality control was performed including internal slide reading and equipment calibration.

### Skin testing

Atopy was assessed by skin prick tests for common aeroallergens (mixture for house dust mite [Dermatophagoides pteronyssinus and Dermatophagoides farinae, grass pollen [cocksfoot and timothy], weed pollen [Parietaria officinalis and Ambrosia artemisifolia], tree pollen [birch, ash tree, olive tree, oak, and cypress], animal danders [cat and dog allergens], and fungal allergens [Aspergillus species and Alternaria alternata]; (Stallergenes, Antony, France) (Montuschi et al., 2006). A positive skin test response was defined as a wheal with a mean diameter (mean of maximum and 90° midpoint diameters) of at least 3 mm greater than that produced with a saline control (Montuschi et al., 2006).

### Multivariate data analysis

E-nose and NMR spectroscopy data analysis requires multivariate statistical algorithms (Bishop, 2006; Wilson and Baietto, 2009). Multilevel PLS was used for data reduction; the K-nearest neighbors method, applied on the multilevel PLS scores, was used for classification purposes (Bishop, 2006; Wilson and Baietto, 2009). The global classification accuracy was assessed by means of Monte Carlo cross-validation scheme. The threshold for significant classification was set at a value of 70% (Bijlsma et al., 2006). Correlations among multidimensional data were calculated using the algorithm implemented in the R-library “psych” (Hahsler et al., 2008; Revelle, 2017) and are shown as a heatmap built using the R-function “heatmap.2,” implemented in the “gplots” package (Warnes et al., 2016).

### Statistical analysis

Data were expressed as mean ± SEM or medians and interquartile ranges (25^th^ and 75^th^ percentiles), after assessing for normality with the D'Agostino-Pearson omnibus normality test. Depending on data distribution, repeated-measures ANOVA or Friedman test was used for assessing within-group pharmacological treatment effect. If overall P value was found significant, paired *t*-test or Wilcoxon signed rank test were performed. Correlation was expressed as a Pearson coefficient. Significance was defined as a value of *P* < 0.05.

In this pilot, proof-of-concept study, we did not adjust our analysis for multiple testing to reduce the risk of missing promising biomarkers, but this also increased our risk of a type I error. Further details on the methodology are provided in the online Supplementary Material (Presentation 1).

## Results

### Study subjects

Subject characteristics are shown in Table 1.

**Table 1:** Subject characteristics^*^.

	**COPD patients (*n* = 14)**
Age, years	73.6 ± 1.8
Gender, females/males	2/14
Pack-years	64.3 ± 8.1
GOLD I/II/III, airflow limitation severity	5/5/4
GOLD A/B/C/D classification	1/7/3/3
^§^Skin prick tests, positive/negative	0/14
History of atopy, positive/negative	0/14
^#^Sputum eosinophils ≥ 3% at visit 1	0/14

* *Data are expressed as numbers or mean ± SEM*.

§ *Common aeroallergens were tested*.

# *Expressed as percentage of total non-squamous cells*.

### Pulmonary function testing

**Figure 2:**
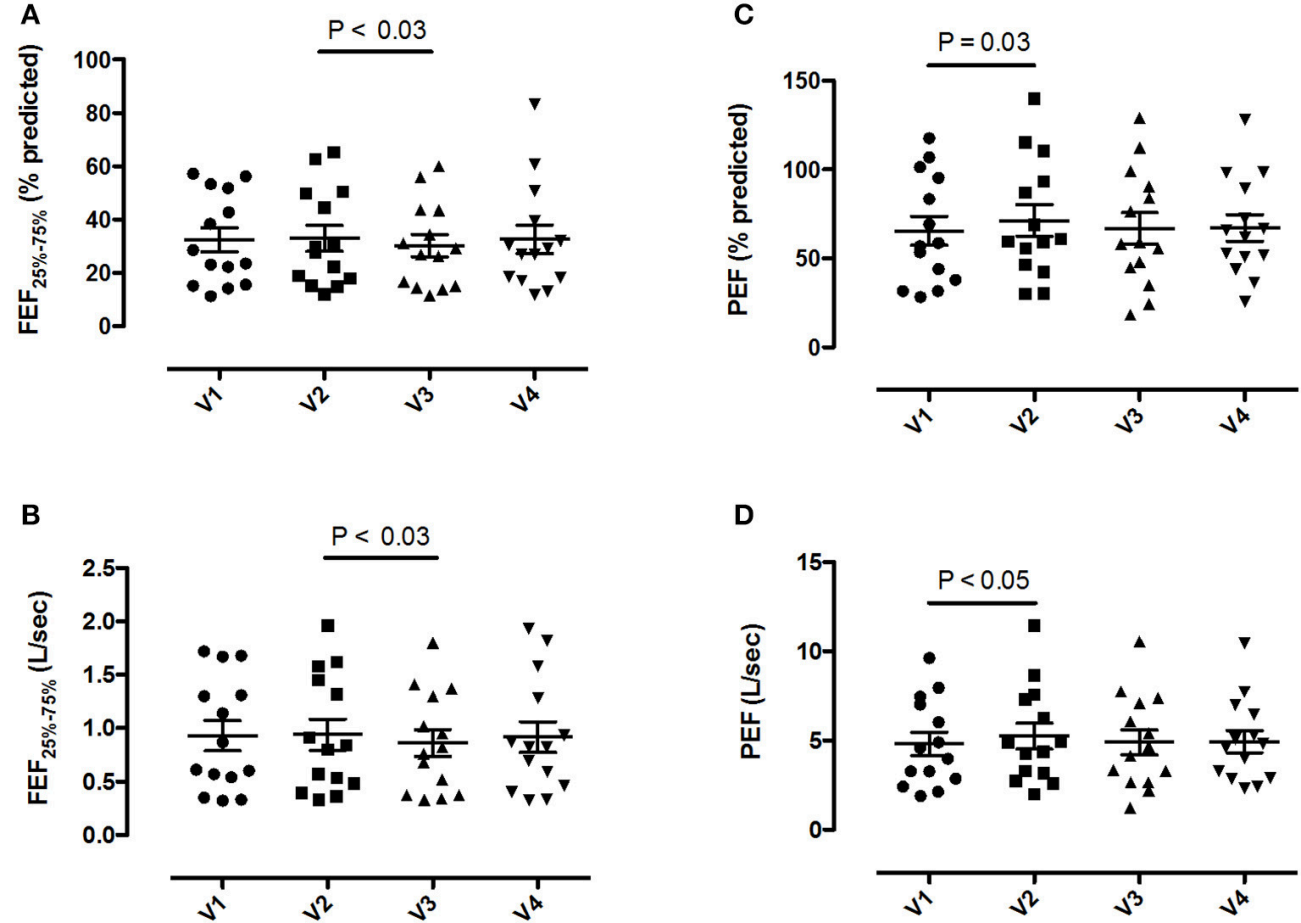
Pre-bronchodilator forced expiratory flow at the 25-75% of the forced vital capacity (FEF_25−75%_) and peak expiratory flow (PEF) values in 14 patients with COPD at visit 1 (V1) to visit 4 (V4). **(A)** FEF_25−75%_ percentage predicted values; **(B)** absolute FEF_25−75%_ values; **(C)** PEF percentage predicted values; **(D)** absolute PEF values. Mean values ± SEM are shown.

Pre-bronchodilator and post-bronchodilator lung function test values across visits in patients with COPD are shown in Tables 2, 3, respectively. Higher mean pre-bronchodilator forced expiratory flow at 25–75% of forced vital capacity (FEF_25−75%_) percentage of predicted and absolute values were observed after 2-week inhaled beclomethasone/formoterol FDC (visit 2) compared with post-4-week treatment with inhaled formoterol alone (visit 3) (*P* = 0.026 and *P* = 0.029, respectively) (Figures 2A,B; Table 2).

**Table 2:** Lung function tests, pre-bronchodilator values^*^.

	**Visit 1 Screening (*n* = 14)**	**Visit 2 Baseline (*n* = 14)**	**Visit 3 Post-withdrawal (*n* = 14)**	**Visit 4 Post-treatment (*n* = 14)**	***P* value**
FEV_1_, L	1.66 ± 0.21	1.68 ± 0.22	1.65 ± 0.21	1.67 ± 0.21	0.907
FEV_1_, % predicted	61.8 ± 6.9	62.7 ± 7.0	61.9 ± 6.7	62.4 ± 6.5	0.962
FVC, L	2.80 ± 0.28	2.86 ± 0.28	2.92 ± 0.28	2.91 ± 0.28	0.440
FVC, % predicted	79.9 ± 6.3	81.8 ± 5.9	84.0 ± 6.0	83.3 ± 5.5	0.534
FEV_1_/FVC, %	58.1 ± 3.2^1^	57.2 ± 3.1	55.3 ± 3.2^1^	56.3 ± 3.3	0.008
PEF, L/s	4.81 ± 0.65^2^	5.24 ± 0.72^2^	4.89 ± 0.70	4.93 ± 0.62	0.044
PEF, % predicted	65.5 ± 8.1^2^	71.3 ± 8.9^2^	66.8 ± 8.8	67.2 ± 7.4	0.033
FEF_25−75%_, L/s	0.92 ± 0.14	0.94 ± 0.15^3^	0.86 ± 0.13^3^	0.92 ± 0.14	0.029
FEF_25−75%_, % predicted	32.4 ± 4.6	32.9 ± 4.9^3^	30.2 ± 4.2^3^	32.6 ± 5.4	0.026

* *Data are expressed as numbers or mean ± SEM. Within-group comparisons were performed with ANOVA for repeated measures and paired t-test. P < 0.05 was considered significant*.

1 V1 vs. V3;

2 V1 vs. V2;

3 *V2 vs. V3*.

*Abbreviations: FEF_25−75%_, forced expiratory flow at 25–75% of the forced vital capacity; FEV_1_, forced expiratory volume in 1 s; FVC, forced vital capacity; PEF, peak expiratory flow*.

**Table 3:** Lung function tests, post-bronchodilator values^*^.

	**Visit 1 (*n* = 14)**	**Visit 2 (*n* = 14)**	**Visit 3 (*n* = 14)**	**Visit 4 (*n* = 14)**	***P*-Value**
FEV_1_, L	1.77 ± 0.22	1.80 ± 0.22	1.79 ± 0.22	1.79 ± 0.22	0.902
FEV_1_, % predicted	66.2 ± 7.0	67.6 ± 7.2	67.0 ± 7.0	66.4 ± 7.0	0.854
FVC, L	2.98 ± 0.28	3.01 ± 0.28	3.07 ± 0.26	3.06 ± 0.28	0.547
FVC, % predicted	85.3 ± 6.2	86.4 ± 6.0	88.7 ± 5.9	87.5 ± 5.6	0.587
FEV_1_/FVC, %	58.3 ± 3.3	58.5 ± 3.3	56.5 ± 3.1	56.7 ± 3.3	0.110
PEF, L/sec	5.38 ± 0.72	5.29 ± 0.64	5.19 ± 0.70	5.12 ± 0.71	0.427
PEF, % predicted	73.1 ± 8.9	72.4 ± 7.9	70.9 ± 8.70	69.4 ± 8.50	0.437
FEF_25−75%_, L/sec	0.99 ± 0.15	1.03 ± 0.16	0.96 ± 0.15	0.96 ± 0.16	0.447
FEF_25−75%_, % predicted	34.7 ± 4.8	36.3 ± 5.2	33.7 ± 4.9	34.0 ± 5.5	0.493

* *Data are expressed as numbers or mean ± SEM. Within-group comparisons were performed with ANOVA for repeated measures and paired t-test. P < 0.05 was considered significant*.

*FEF_25−75%_, forced expiratory flow at 25–75% of the forced vital capacity; FEV_1_, forced expiratory volume in 1 s; FVC, forced vital capacity; PEF, peak expiratory flow*.

Mean pre-bronchodilator FEV_1_/FVC ratio was higher on maintenance treatment with fluticasone propionate/salmeterol FDC (visit 1) compared with post-treatment with formoterol alone (visit 3) (*P* = 0.008) (Table 2).

Higher mean pre-bronchodilator peak expiratory flow (PEF) percentage of predicted and absolute values were observed after 2-week beclomethasone/formoterol FDC (visit 2) compared with maintenance treatment with inhaled fluticasone propionate/salmeterol (visit 1) (*P* = 0.033 and *P* = 0.044, respectively) (Figures 2C,D, Table 2). No within-group differences in post-bronchodilator functional parameters were observed (Table 3).

### Electronic nose

E-nose analysis with either e-nose was successfully performed in all 14 subjects at all visits. A total of 56 breathprints for each e-nose was collected. Fifteen out of 32 carbon polymer sensors showed significant mean differences between inhaled fluticasone propionate/salmeterol FDC maintenance treatment (visit 1) and post-4-week beclomethasone/formoterol FDC treatment (visit 4), whereas 7 sensors showed significant mean differences between fluticasone propionate/salmeterol maintenance treatment (visit 1) and 2-week treatment with beclomethasone/formoterol (visit 2) (Table 4).

**Table 4:** Univariate analysis of carbon polymer sensor e-nose response^*^.

**V1**	**V2**	**V3**	**V4**	***P*-value**
**Sensor 1**				
2.507 ± 0.2171	2.843 ± 0.1636	2.891 ± 0.2411	3.208 ± 0.1857	0.0480
V1 vs. V4				0.0055
**Sensor 2**				
1.664 ± 0.1427	1.891 ± 0.0979	1.934 ± 0.1537	2.181 ± 0.1260	0.0212
V1 vs. V2				0.0364
V1 vs. V4				0.0053
Sensor 3				
2.527 ± 0.2057	2.768 ± 0.1549	2.868 ± 0.2432	3.183 ± 0.1993	n.s.
**Sensor 4**				
1.447 ± 0.1191	1.640 ± 0.0891	1.693 ± 0.1378	1.926 ± 0.1249	0.0209
V1 vs. V4				0.0040
Sensor 5				
11.27 ± 1.195	12.86 ± 0.8729	13.38 ± 1.261	16.70 ± 2.785	n.s.
Sensor 6				
6.574 ± 0.7314	7.547 ± 0.5477	7.757 ± 0.8441	8.510 ± 0.6821	n.s.
**Sensor 7**				
0.001109 ± 0.0962	0.001253 ± 0.0678	0.001265 ± 0.1008	0.001409 ± 0.0763	0.0409
V1 vs. V4				0.0053
**Sensor 8**				
0.6803 ± 0.05038	0.7709 ± 0.03499	0.7732 ± 0.05597	0.8569 ± 0.05064	0.0259
V1 vs. V2				0.0216
V1 vs. V4				0.0058
Sensor 9				
2.154 ± 0.2058	2.462 ± 0.1496	2.542 ± 0.2280	2.811 ± 0.1892	n.s.
Sensor 10				
1.283 ± 0.1128	1.474 ± 0.08070	1.492 ± 0.1259	1.649 ± 0.1020	n.s.
Sensor 11				
1.277 ± 0.1155	1.429 ± 0.0759	1.460 ± 0.1267	1.607 ± 0.0973	n.s.
**Sensor 12**				
1.321 ± 0.1193	1.487 ± 0.0781	1.559 ± 0.1282	1.715 ± 0.0970	0.0300
V1 vs. V4				0.0046
Sensor 13				
1.036 ± 0.0877	1.185 ± 0.0624	1.196 ± 0.0981	1.315 ± 0.0757	n.s.
**Sensor 14**				
0.8994 ± 0.0752	1.014 ± 0.0542	1.048 ± 0.0820	1.163 ± 0.0604	0.0258
V1 vs. V4				0.0051
**Sensor 15**				
2.225 ± 0.1933	2.513 ± 0.1338	2.577 ± 0.2234	2.881 ± 0.1799	0.045
V1 vs. V4				0.0071
**Sensor 16**				
1.241 ± 0.1085	1.405 ± 0.0809	1.447 ± 0.1203	1.577 ± 0.0962	0.0100
V1 vs. V4				0.0094
Sensor 17				
1.430 ± 0.1237	1.603 ± 0.0880	1.637 ± 0.1437	1.804 ± 0.1222	n.s.
Sensor 18				
2.643 ± 0.2480	3.000 ± 0.1781	3.093 ± 0.2729	3.385 ± 0.2228	n.s.
**Sensor 19**				
0.9070 ± 0.0727	1.033 ± 0.0569	1.075 ± 0.0908	1.172 ± 0.0648	0.0273
V1 vs. V2				0.0320
V1 vs. V4				0.0052
Sensor 20				
2.341 ± 0.2267	2.658 ± 0.1648	2.702 ± 0.2400	2.910 ± 0.1990	n.s.
**Sensor 21**				
0.8288 ± 0.0683	0.9423 ± 0.0510	0.9509 ± 0.0762	1.057 ± 0.0597	0.0362
V1 vs. V2				0.0307
V1 vs. V4				0.0062
Sensor 22				
1.004 ± 0.0863	1.132 ± 0.0594	1.132 ± 0.0935	1.233 ± 0.0767	n.s.
Sensor 23				
9.586 ± 0.9759	10.94 ± 0.7091	11.47 ± 1.157	13.26 ± 1.443	n.s.
**Sensor 24**				
1.403 ± 0.1185	1.597 ± 0.0943	1.704 ± 0.1439	1.904 ± 0.0872	0.0084
V1 vs. V2				0.0404
V1 vs. V4				0.0030
V2 vs. V4				0.0365
**Sensor 25**				
1.426 ± 0.1198	1.618 ± 0.0865	1.635 ± 0.1300	1.849 ± 0.1003	0.0286
V1 vs. V2				0.0494
V1 vs. V4				0.0051
Sensor 26				
0.003672 ± 0.3577	0.004201 ± 0.2878	0.004370 ± 0.4194	0.004926 ± 0.4143	n.s.
**Sensor 27**				
0.0005989 ± 0.0456	0.0006938 ± 0.0359	0.0007088 ± 0.0597	0.0007838 ± 0.0412	0.0182
V1 vs. V2				0.0170
V1 vs. V4				0.0013
Sensor 28				
0.004238 ± 0.4047	0.004810 ± 0.2025	0.004937 ± 0.4320	0.005434 ± 0.3809	n.s.
Sensor 29				
2.228 ± 0.2167	2.521 ± 0.1581	2.573 ± 0.2285	2.890 ± 0.2030	n.s.
Sensor 30				
0.9987 ± 0.0863	1.151 ± 0.0666	1.170 ± 0.0987	1.301 ± 0.0946	n.s.
Sensor 31				
15.13 ± 1.761	17.41 ± 1.240	18.35 ± 2.133	24.58 ± 6.076	n.s.
**Sensor 32**				
0.6599 ± 0.0538	0.7333 ± 0.0397	0.7486 ± 0.0599	0.8363 ± 0.0437	0.0426
V1 vs. V4				0.0066

*The behavior of each sensor (sensor 1–sensor 32) is shown. Sensors whose mean values differ across visits are shown in bold*.

* *Data, expressed as mean ± SEM, are relative changes in sensor resistance [(Rmax-R0)/R0] (μOhms). Intra-group, between-visit comparisons were performed with ANOVA for repeated measures. If overall P was lower than 0.05, considered significant, paired t-test was performed. n.s., not significant; V, visit*.

Consistent with carbon polymer sensor behavior, two quartz crystal sensors showed significant mean differences between fluticasone propionate/salmeterol maintenance treatment (visit 1) and post-4-week beclomethasone/formoterol FDC treatment (visit 4); sensor 3 and 4 showed significant mean differences between fluticasone propionate/salmeterol maintenance treatment (visit 1) and 2-week beclomethasone/formoterol treatment (visit 2) (Table 5).

**Table 5:** Univariate analysis of quartz crystal sensor e-nose response^*^.

**V1**	**V2**	**V3**	**V4**	***P***
Sensor 1				
21.5 (14.75–31)	30 (19–38)	27.5 (21.75–36.75)	30.5 (22.75–38.25)	n.s.
**Sensor 2**				
19 (14–29)	27.5 (20.5–32.75)	25 (21.5–35.25)	30.5 (25.5–38)	0.0092
V1 vs. V4				0.0329
**Sensor 3**				
49 (38.25–76.25)	79.5 (57–105.8)	66.5 (53.5–106.5)	89 (65.5–101)	n.s.
V1 vs. V2				0.0279
**Sensor 4**				
31 (26.75–48)	48.5 (37.25–58.25)	44 (36–66.75)	54.4 (43.5–67)	0.0523
V1 vs. V2				0.0445
V1 vs. V4				0.0413
Sensor 5				
12.5 (9–16.8)	19 (13.3–22.5)	15.5 (12.5–25.5)	19.5 (15.8–24.3)	n.s.
Sensor 6				
24 (18.8–33.3)	29.5 (23.3–43.8)	36 (25–41)	32 (24.8–45)	n.s.
Sensor 7				
20.5 (15.3–32)	29 (22.3–38)	29 (25.8–41.3)	32.5 (29–36.8)	n.s.
Sensor 8				
30.5 (20.3–44.5)	42 (32.5–55.3)	30 (27.5–48.3)	41.5 (29.5–49.5)	n.s.

*The behavior of each sensor (sensor 1–sensor 8) is shown. Sensors whose mean values differ across visits are shown in bold*.

* *Data, expressed as median and interquartile range, are relative changes in sensor frequency [(Rmax-R0)/R0] (Hz). Intra-group, between-visit comparisons were performed with Friedman's test. If overall P was lower than 0.05, considered significant, Wilcoxon signed rank test was performed. n.s., not significant; V, visit*.

There was no difference in sensor response using either e-nose when other paired visits were compared (Tables 4, 5).

### Metabolomic analysis of EBC with NMR spectroscopy

**Figure 3:**
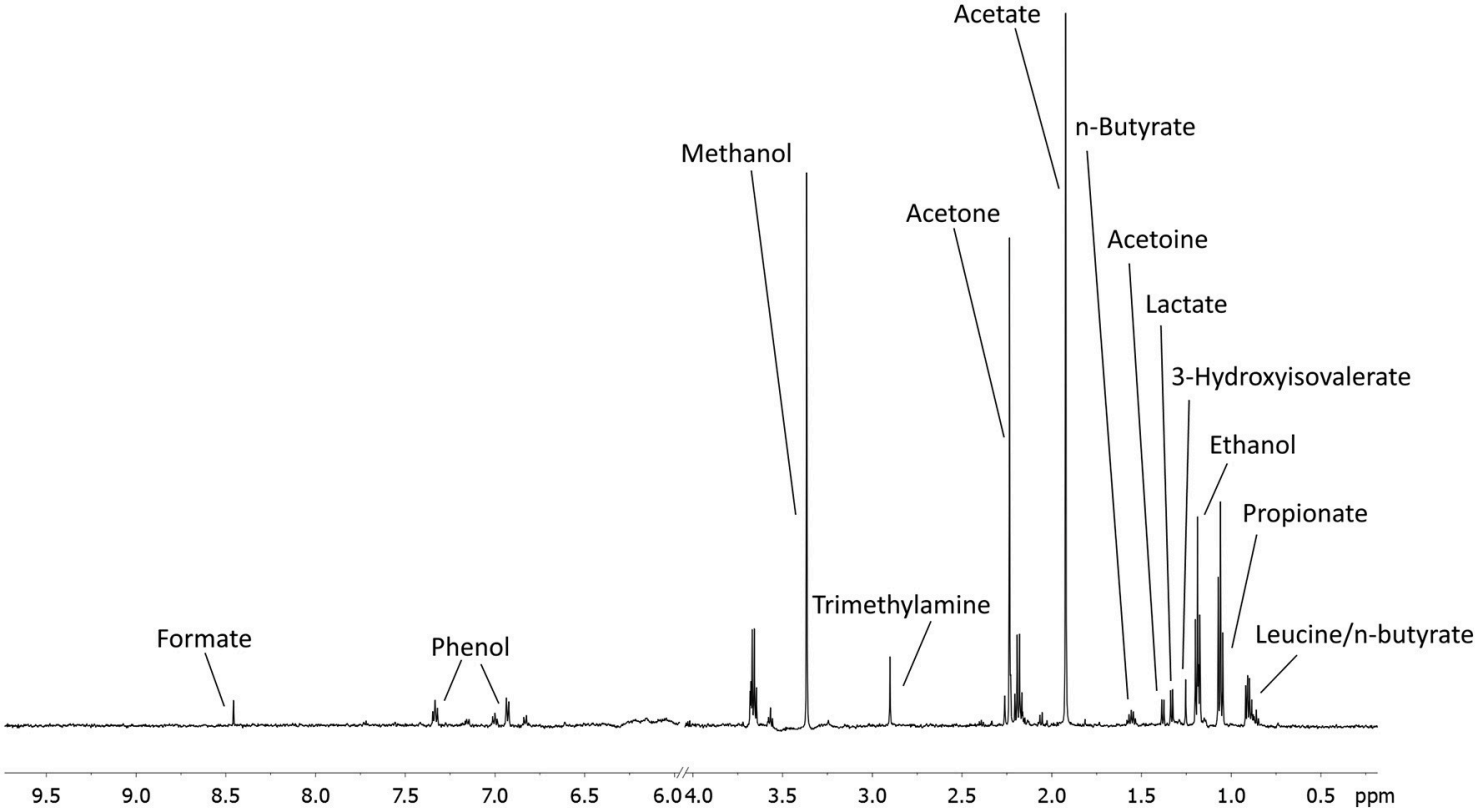
An example of a typical EBC NMR spectrum at 600 MHz. All metabolites assigned and quantified are reported in figure.

A total of 56 EBC NMR spectra obtained from 14 study subjects across visits were analyzed. Typical EBC ^1^H-NMR spectra obtained from a COPD patient across visits are shown in Figure S1. EBC metabolomics with NMR spectroscopy discriminated between fluticasone propionate/salmeterol FDC maintenance treatment (visit 1) and treatment with beclomethasone/formoterol FDC for 4 weeks (visit 4) (accuracy = 72%, *P* = 0.01) (Figure S2). Formate levels were higher at visit 1 (694.9 ± 360.5 arbitrary units, median ± median absolute deviation [MAD]) than at visit 4 (409.2 ± 224.8 arbitrary units, *P* = 0.029) (Table 6, Figures S3A,B). Other paired comparisons showed accuracy below the significant threshold set at 70% (Table S1). After 4-week treatment with beclomethasone/formoterol FDC (visit 4), EBC acetate levels were lower than those measured after 4-week treatment with formoterol alone (visit 3) (*P* = 0.009) (Table 6). Several EBC metabolites, including formate, phenol, methanol, trimethylamine, acetone, acetoine, acetate, n-butyrate, lactate, 3-hydroxyisovalerate, ethanol, propionate, and leucine-o-butyrate were identified (Figure 3). Apart from formate and acetate, their levels were similar across visits (Table 6).

**Table 6:** Paired comparison of exhaled breath condensate (EBC) metabolite concentrations at each visit using Wilcoxon signed-rank test.

	**Visit 1**	**Visit 2**	**Visit 3**	**Visit 4**	**Visit 1 vs. visit 2**	**Visit 1 vs. visit 3**	**Visit 1 vs. visit 4**	**Visit 2 vs. visit 3**	**Visit 2 vs. visit 4**	**Visit 3 vs. visit 4**
**Metabolite**	**Median ± MAD**	**Median ± MAD**	**Median ± MAD**	**Median ± MAD**	***P***	***P***	***P***	***P***	***P***	***P***
Acetate	7, 322.51 ± 3, 372.29	8, 634.57 ± 6, 598.25	10, 385.09 ± 4, 929.83	4, 683.2 ± 1, 518.54	0.95	0.33	0.33	0.95	0.12	0.01
Acetoine	706.10 ± 482.55	512.18 ± 215.73	730.90 ± 311.39	546.27 ± 360.24	0.12	0.54	0.27	0.09	0.54	0.12
Acetone	6, 152.10 ± 3, 456.11	6, 072.73 ± 1, 730.04	6, 850.06 ± 2, 420.90	6, 579.12 ± 2, 468.53	0.81	1.00	0.22	0.58	0.63	0.17
3-Hydroxyisovalerate	326.31 ± 64.86	351.19 ± 45.66	388.33 ± 44.43	376.95 ± 89.09	0.71	0.30	0.58	0.33	0.81	0.33
Ethanol	488.16 ± 99.84	538.82 ± 201.68	754.17 ± 384.75	544.54 ± 238.66	1.00	0.06	0.95	0.39	0.95	0.08
Formate	694.95 ± 360.47	695.14 ± 519.50	424.52 ± 131.84	409.16 ± 224.76	0.86	0.67	0.03	0.17	0.05	0.05
Lactate	444.24 ± 158.85	284.10 ± 92.29	352.78 ± 142.45	289.46 ± 115.33	0.95	0.95	0.12	0.95	0.12	0.30
Leucine/n-butyrate	1, 431.78 ± 482.18	2, 484.15 ± 1743.00	2, 002.49 ± 896.17	1, 583.98 ± 537.93	0.50	0.12	0.67	0.71	0.12	0.06
Methanol	14, 225.48 ± 7, 044.87	16, 268.66 ± 7, 711.03	11, 570.77 ± 2, 751.26	14, 358.75 ± 8, 341.86	1.00	0.36	0.95	0.81	0.63	0.67
n-butyrate	664.07 ± 198.96	1, 572.65 ± 1, 205.33	1, 099.32 ± 468.94	766.91 ± 251.52	0.22	0.14	0.86	0.71	0.12	0.17
Phenol	443.05 ± 224.90	325.68 ± 204.11	354.39 ± 158.40	335.79 ± 204.24	0.22	0.76	0.36	0.07	0.63	0.39
Proprionate	1, 542.86 ± 739.74	2, 394.86 ± 1, 967.45	2, 519.65 ± 1, 754.52	988.23 ± 706.23	0.46	0.12	0.58	0.46	0.46	0.09
Trimethylamine	364.99 ± 260.65	343.87 ± 262.45	348.39 ± 210.49	301.78 ± 195.88	0.76	0.36	0.86	0.39	0.90	0.46

*Metabolite concentrations are reported as median arbitrary units ± median absolute deviation (MAD)*.

### F_E_NO

There was no difference in F_E_NO concentrations in patients with COPD across visits (overall *P* = 0.35) (Table S2).

### Measurement of PGE_2_ and 15-F_2t_-isoprostane in sputum supernatants and EBC

PGE_2_ concentrations were detected in 49 out of a total of 56 sputum supernatant samples. Compared with formoterol alone post-treatment values (visit 3), lower sputum PGE_2_ concentrations were observed after 4-week treatment with beclomethasone/formoterol FDC (visit 4) (*P* = 0.008) and on maintenance treatment with fluticasone propionate/salmeterol FDC (visit 1) (*P* = 0.021) (Table S3). These data suggest that treatment with ICS FDC, containing either fluticasone propionate or beclomethasone dipropionate, reduces sputum PGE_2_ concentrations. There was no between-visit differences in EBC PGE_2_ or sputum and EBC 15-F_2t_-isoprostane concentrations (Table S3).

### Sputum cell analysis

Eight patients with COPD had a complete set of sputum slides (visit 1 to visit 4) (Table S4). No patient with COPD had sputum eosinophilia, as defined by sputum cell counts >3%, at visit 1 (screening visit). There was no within-group difference in sputum cell counts (Table S4). Percentage sputum cell counts in all valid sputum slides are shown in Table S5.

### Correlations

**Figure 4:**
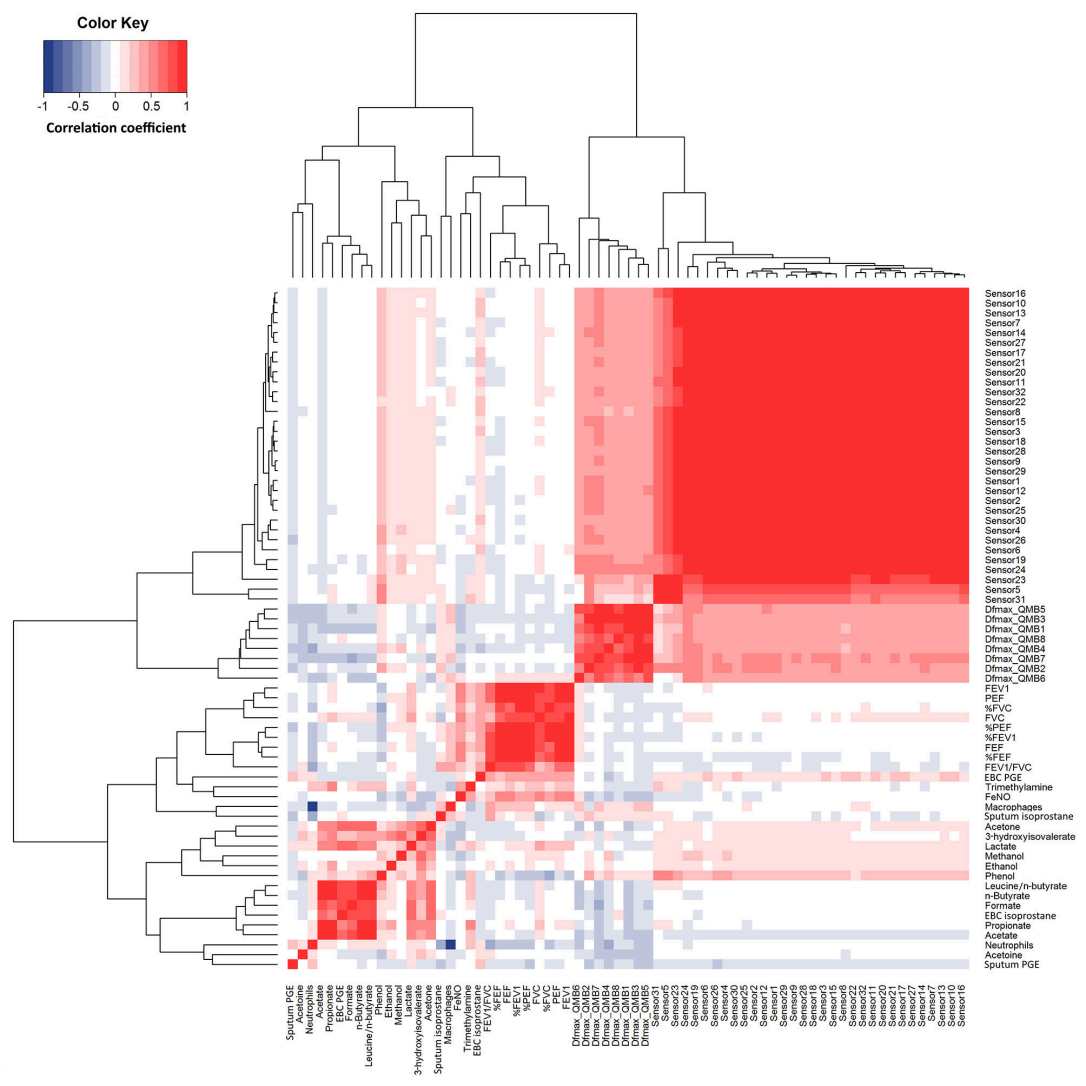
Heatmap showing correlations between study outcome measures in 14 patients with COPD at visit 1 to visit 4 (*n* = 56). R values are shown as different degree of color intensity (red, positive correlations; blue, negative correlation).

Responses within each individual e-nose and between e-noses were correlated, whereas EBC metabolites detected by NMR spectroscopy were not correlated with either e-noses, except EBC phenol which was correlated with 16 carbon polymer sensors (Figure 4). In EBC, there was a correlation between PGE_2_ and formate (*r* = 0.78, *P* = 5.06·10^−11^), acetone (*r* = 0.63, *P* = 1.64·10^−6^), lactate (*r* = 0.64, *P* = 6.03·10^−7^), n-butyrate (*r* = 0.82, *P* = 5.89·10^−13^), propionate (*r* = 0.59, *P* = 8.26·10^−6^), and acetate (*r* = 0.71, *P* = 1.23·10^−8^) (Figure 4).

Quartz crystal sensor 4 (*r* = −0.35, *P* = 0.023) and 7 (*r* = −0.31, *P* = 0.041) negatively correlated with sputum neutrophils. These showed negative correlation with FEV_1_/FVC (*r* = −0.34; *P* = 0.028) (Figure 4).

### Multidimensional integrated model for assessment of pharmacological treatment

In the 14 COPD study participants, multidimensional pairwise discrimination models were built. The models discriminated between maintenance treatment with fluticasone propionate/salmeterol (visit 1) vs. 4-week treatment with formoterol alone (visit 3) (accuracy = 71.5%, *P* < 0.01); the univariate analysis showed differences in sputum supernatant PGE_2_ and FEV_1_/FVC ratio (Table 7); between maintenance treatment with fluticasone propionate/salmeterol (visit 1) vs. 4-week treatment with beclomethasone/formoterol (visit 4) (accuracy = 82.5%, *P* < 0.01); the univariate analysis showed differences in EBC formate and e-noses (Table 7); between 4-week treatment with formoterol alone (visit 3) vs. 4-week treatment with beclomethasone/formoterol (visit 4) (accuracy = 74.6%, *P* < 0.01); the univariate analysis showed differences in sputum PGE_2_ and EBC acetate (Table 7).

**Table 7:** Classification accuracies with 95% confidence interval and P values among different pharmacological treatments from visit 1 to visit 4 based on multidimensional PLS models in 14 patients with COPD.

**Comparison**	**Overall accuracy (*P*-value)**	**Model quality**	**Variable**		***P*-value**
Visit 1 vs. Visit 2	66.4% (CI 95% 66.0–66.9%) (*P* = 0.12)	AUC 0.714 Youden's J 0.294	Carbon polymer sensor e-nose	Sensor2	0.036
				Sensor8	0.021
				Sensor19	0.032
				Sensor21	0.031
				Sensor24	0.040
				Sensor25	0.049
				Sensor27	0.017
			Quartz crystal sensor e-nose	Sensor 3	0.028
				Sensor 4	0.044
			Spirometry	PEF, % pred	0.033
				PEF, L	0.044
Visit 1 vs. Visit 3	71.5% (CI 95% 71.1–72.0%) (*P* < 0.01)	AUC 0.704 Youden's J 0.446	Eicosanoids	PGE_2_ in sputum supernatants	0.021
			Spirometry	FEV_1_/FVC, %	0.008
Visit 1 vs. Visit 4	82.5% (CI 95% 82.0–83.0%) (*P* < 0.01)	AUC 0.857 Youden's J 0.666	NMR spectroscopy	Formate in EBC	0.029
			Carbon polymer sensor e-nose	Sensor1	0.006
				Sensor2	0.005
				Sensor4	0.004
				Sensor7	0.005
				Sensor8	0.006
				Sensor12	0.005
				Sensor14	0.005
				Sensor15	0.007
				Sensor16	0.009
				Sensor19	0.005
				Sensor21	0.006
				Sensor24	0.003
				Sensor25	0.005
				Sensor27	0.001
				Sensor32	0.007
			Quartz crystal sensor e-nose	Sensor 2	0.032
				Sensor 4	0.041
Visit 2 vs. Visit 3	59.9% (CI 95% 58.8–61.1%) (*P* = 0.23)	AUC 0.571 Youden's J 0.134	Spirometry	FEF_25−75%_, L	0.029
				FEF_25−75%_, % pred	0.026
Visit 2 vs. Visit 4	59.8% (CI 95% 58.2–61.3%) (*P* = 0.12)	AUC 0.556 Youden's J 0.214	Carbon polymer sensor e-nose	Sensor 24	0.037
Visit 3 vs. Visit 4	74.6% (CI 95% 73.9–75.2%) (*P* < 0.01)	AUC 0.760 Youden's J 0.454	Eicosanoids	PGE_2_ in sputum supernatants	0.008
			NMR spectroscopy	Acetate in EBC	0.009

*The significant variables in the univariate analysis of each experimental technique are also reported (Wilcoxon signed rank test P-values). AUC, area under the curve; EBC, exhaled breath condensate; FEF_25−75%_, forced expiratory flow at 25–75% of the forced vital capacity; FEV_1_, forced expiratory volume in 1 s; FVC, forced vital capacity; NMR, nuclear magnetic resonance; PEF, peak expiratory flow; PGE_2_, prostaglandin E_2_; % pred, percentage of predicted value; PLS, partial least squares*.

The multidimensional models showed higher accuracy than the models based on spirometry alone (Table 8). PLS score plots are centrosymmetric given the pairwise nature of the analyses performed which leads to a matrix with a two block structure with opposite signs, thus producing symmetric PLS scores. This explains the same values of sensitivity and specificity. During cross-validation, when a multilevel PLS model is built from the training set, the entire variation splitting procedure is performed (van Velzen et al., 2008; Westerhuis et al., 2010). The procedure should, therefore, be adapted to keep the paired data structure both in the training and in the test set. As a result, complete individuals are left out of the training set (per individual validation, not per sample). At each step, if a sample of one individual is mistaken, inevitably the other is mistaken in the opposite way, leading to a fully symmetric confusion matrix. Of course, this symmetry is broken when considering more than just two time points. Further information on results is provided in the online Supplementary Material (Presentation 1).

**Table 8:** Comparison between a multidimensional integrated model including breathomics and a model based on spirometry alone used for assessing the effects of pharmacological treatment in 14 patients with COPD.

**Comparison**	**Spirometry-based model**					**Multidimensional integrated model**				
	**AUC**	*P*-value^*^	**OR**	**Sensitivity**	**Specificity**	**AUC**	*P*-value^*^	**OR**	**Sensitivity**	**Specificity**
Visit 1 vs. Visit 2	0.689	*P* = 0.06	3.45	0.650	0.650	0.714	*P* = 0.12	3.90	0.664	0.664
Visit 1 vs. Visit 3	0.704	*P* = 0.07	3.54	0.653	0.653	0.704	*P* < 0.01	6.29	0.715	0.715
Visit 1 vs. Visit 4	0.561	*P* = 0.90	0.34	0.367	0.367	0.857	*P* < 0.01	22.2	0.825	0.825
Visit 2 vs. Visit 3	0.729	*P* = 0.03	6.36	0.716	0.716	0.571	*P* = 0.23	2.23	0.599	0.599
Visit 2 vs. Visit 4	0.648	*P* = 0.43	0.85	0.480	0.480	0.556	*P* = 0.12	2.21	0.598	0.598
Visit 3 vs. Visit 4	0.658	*P* = 0.11	3.16	0.640	0.640	0.760	*P* < 0.01	8.63	0.746	0.746

*AUC, area under the curve; OR, odds ratio*.

* *P-values have been obtained through 100-fold permutation tests and are referred to the AUC values*.

## Discussion

The principal messages of the present study are: (1) breathomics can be successfully applied to assessment of effects of corticosteroid treatment and withdrawal with ICS/LABA in patients with COPD; (2) breathomics results are confirmed by the concordance of three different breathomics techniques (carbon polymer sensor e-nose, quartz crystal sensor e-nose, NMR-based metabolomics) applied to the gaseous and aerosol particle (EBC) phase of the exhaled breath. These techniques provide complementary information; (3) a multidimensional, integrated, model including breathomics, improves the ability of identifying pharmacological treatment-induced effects compared with a monodimensional model based on standard pulmonary function testing; (4) this approach provides insights into the anti-inflammatory effects of ICS in patients with COPD as reflected by reduced sputum PGE_2_ and EBC acetate concentrations after beclomethasone/formoterol FDC vs. formoterol alone.

In a breathomics multidimensional approach to assessment of drugs for COPD, we show anti-inflammatory effects of an extrafine beclomethasone/formoterol FDC pMDI vs. formoterol alone as reflected by reduced levels of sputum PGE_2_, a potent inflammatory mediator in the airways (Clarke et al., 2005; Holden et al., 2010), and EBC acetate which were found elevated in COPD patients (De Laurentiis et al., 2008, 2013; Airoldi et al., 2016; Santini et al., 2016).

We also show that short-term treatment with an extrafine beclomethasone/formoterol FDC pMDI is associated with different breathprints as compared with regular fluticasone propionate/formoterol FDC DPI in patients with COPD. These results might suggest that various ICS/LABA formulations have different effects on breathomics outcomes, although the biological implications of these findings are unknown and have to be defined.

Treatment with either ICS/LABA FDC vs. inhaled formoterol alone was associated with a slight, but significant, increase in small airway function as reflected by FEF_25−75%_ and FEV_1_/FVC values, whereas higher PEF values were observed after beclomethasone/formoterol (visit 2) than on regular fluticasone propionate/salmeterol (visit 1), suggesting that treatment-induced e-nose breathprint variations parallel the observed functional changes only to a limited extent. Interestingly, functional effects were observed after only 4-week treatment with inhaled beclomethasone/formoterol, a relatively short duration of treatment for COPD trials, and in patients with COPD who had normal sputum eosinophils, negative reversibility test to bronchodilators, negative skin prick tests, and no history of atopy, thus, excluding an asthma component, on which ICS are generally more effective.

The strong correlation between most sensors within each individual e-nose indicates a high degree of sensor redundancy, whereas a similar behavior of e-nose based on different technologies confirms the results of the exhaled breath analysis. Of note, quartz crystal sensors, but not carbon polymer sensors, showed correlation with sputum neutrophils. On the other hand, the lack of correlation between EBC metabolites detected by NMR spectroscopy and either e-noses suggests that a comprehensive breathomics approach might be complementary and increase the level of information. In EBC, there was a correlation between PGE_2_, an eicosanoid which can have potent pro-inflammatory effects in the airways, and formate (*r* = 0.78), acetone (*r* = 0.63), lactate (*r* = 0.64), n-butyrate (*r* = 0.82), propionate (*r* = 0.59), and acetate (*r* = 0.71) suggesting that these EBC metabolites might reflect respiratory inflammation. These correlations are unlikely to be explained by individual variability in aerosol particle formation (Effros et al., 2003) as there was no correlation between EBC 15-F_2t_-isoprostane and EBC metabolites nor between EBC PGE_2_ and EBC 15-F_2t_-isoprostane. For discussion on correlations see also online Supplemenatry Material (Presentation 1).

Unlike a standard efficacy model based on spirometry, the multidimensional model used in our study was able to distinguish between pharmacological treatments (accuracy > 70%) in 3 out of 6 possible paired comparisons. This might increase the chance of detecting drug effects in COPD patients. In line with an anti-inflammatory effect of ICS, sputum concentrations of PGE_2_, the key between-treatment discriminating outcome measure, were lower after treatment with either ICS/LABA FDC compared with formoterol alone. By contrast, other inflammatory outcomes, including sputum neutrophil cell counts, EBC PGE_2_, and sputum and EBC 15-F_2t_-isoprostane, showed steroid resistance.

Most of the EBC metabolites derive from pyruvate (Airoldi et al., 2016). Many breath volatile and non-volatile compounds are product of bacterial metabolism (Airoldi et al., 2016). Moreover, breathomics techniques, including e-noses and NMR spectroscopy, can be used for detecting and identifying bacterial species (Lim et al., 2016; Palama et al., 2016). For these reasons, exhaled breath analysis has been proposed as a powerful tool to identify bacterial metabolomic signatures (Airoldi et al., 2016). However, our approach is not suitable for identifying the cellular source(s) of EBC metabolites for which *in vitro* studies are required.

Elevated EBC formate and acetate levels have been reported in COPD patients compared with healthy subjects (De Laurentiis et al., 2008, 2013). These findings have been confirmed in a recent ^1^H-NMR spectroscopy study showing that EBC acetate is 36-fold higher and EBC formate is 2.5 higher in patients with emphysema due to α_1_-antitrypsin deficiency than in healthy subjects. Interestingly, we found reduced EBC levels of acetate and formate after 4-week beclomethasone/formoterol treatment. These preliminary findings suggest a potential anti-inflammatory mechanism of ICS involving a change in bacterial metabolism which might have profound implications in how ICS reduce the frequency of COPD exacerbations or increase the risk of pneumonia.

Study strengths rely on the fact that this is the first prospective evidence of the effects of pharmacological treatment and steroid withdrawal on breathomics in COPD patients, the multidimensional, non-invasive, approach to drug assessment requiring a systems medicine-based data analysis, the use of complementary breathomics techniques, and the completeness of data collected. The limited number of study subjects, the open-label, uncontrolled, design of the pharmacological study, the short duration of treatment/withdrawal phases, the lack of training and testing validation and external validation cohorts, represent limitations which preclude definitive conclusions.

In conclusion, breathomics can be used for assessing the effects of treatment and steroid withdrawal with ICS/LABA in patients with COPD. The present pilot, proof-of-concept, study provides a rational basis for large, randomized, controlled, pharmacological trials in patients with COPD using a similar multidimensional approach.

## Guarantor statement

PM takes responsibility for the content of the manuscript and the work as a whole, including the integrity of data and data analysis.

## Author contributions

PM was responsible for study planning, study design, clinical trial, electronic nose analysis, data analysis, data interpretation, and manuscript writing. GS was responsible for measurement of F_E_NO, spirometry, sputum induction and analysis, data analysis, and manuscript revision. NM was responsible for study planning, sputum induction and analysis, data analysis, data interpretation, drug vigilance, and manuscript revision. AV was responsible for EBC NMR spectroscopy, multivariate data analysis, data interpretation, and manuscript revision. FM was responsible for patient recruitment, spirometry and manuscript revision. RS was responsible for measurement of PGE_2_ and 15-F_2t_-isoprostane in sputum supernatants and EBC, and for manuscript revision. LT was responsible for EBC NMR spectroscopy, multivariate data analysis, data interpretation, and manuscript revision. GZ was responsible for sputum cell analysis and manuscript revision. LF was responsible for patient recruitment, spirometry and manuscript revision. CM was responsible for skin prick testing and manuscript revision. CDN was responsible for e-nose prototype design, chemical sensor assembling and instrument calibration, and manuscript revision. AD was responsible for e-nose prototype design, chemical sensor assembling and instrument calibration, and manuscript revision. CL was responsible for EBC NMR spectroscopy, data analysis, multivariate data analysis, data interpretation, and manuscript preparation and revision. PB was responsible for manuscript preparation and revision. TH was responsible for study planning, study design, data interpretation, and manuscript preparation and revision.

### Conflict of interest statement

TH, a full time employee of Allergy Therapeutics (UK) Ltd. The other authors declare that the research was conducted in the absence of any commercial or financial relationships that could be construed as a potential conflict of interest. The handling editor is currently co-organizing a Research Topic with one of the authors PM, and confirms the absence of any other collaboration.

## References

[B1] Airoldi C. Ciaramelli C. Fumagalli M. Bussei R. Mazzoni V. Viglio S.. (2016). ^1^H NMR to explore the metabolome of exhaled breath condensate in α_1_-antitrypsin deficient patients: a pilot study. J. Proteome Res. 15, 4569–4578. 10.1021/acs.jproteome.6b00648 27646345

[B2] American Thoracic Society and European Respiratory Society (2005). ATS/ERS recommendations for standardized procedures for the online and offline measurement of exhaled lower respiratory nitric oxide and nasal nitric oxide, 2005. Am. J. Respir. Crit. Care Med. 171, 912–930. 10.1164/rccm.200406-710ST 15817806

[B3] Barnes P. J. (2010). Inhaled corticosteroids in COPD: a controversy. Respiration 80, 89–95. 10.1159/000315416 20501985

[B4] Bertini I. Luchinat C. Miniati M. Monti S. Tenori L. (2014). Phenotyping COPD by ^1^H NMR metabolomics of exhaled breath condensate. Metabolomics 10, 302–311. 10.1007/s11306-013-0572-3

[B5] Bijlsma S. Bobeldijk I. Verheij E. R. Ramaker R. Kochhar S. Macdonald I. A.. (2006). Large-scale human metabolomics studies: a strategy for data (pre-) processing and validation. Anal. Chem. 78, 567–574. 10.1021/ac051495j 16408941

[B6] Bishop C. M. (2006). Pattern Recognition and Machine Learning. New York, NY: Springer-Verlag.

[B7] Bofan M. Mores N. Baron M. Dabrowska M. Valente S. Schmid M.. (2013). Within-day and between-day repeatability of measurements with an electronic nose in patients with COPD. J. Breath Res. 7:017103. 10.1088/1752-7155/7/1/017103 23445725

[B8] Clarke D. L. Belvisi M. G. Smith S. J. Hardaker E. Yacoub M. H. Meja K. K.A.. (2005). Prostanoid receptor expression by human airway smooth muscle cells and regulation of the secretion of granulocyte colony-stimulating factor. Am. J. Physiol. Lung Cell Mol. Physiol., 288, L238–L250. 10.1152/ajplung.00313.2004 15640521

[B9] De Laurentiis G. Paris D. Melck D. Maniscalco M. Marsico S. Corso G.. (2008). Metabonomic analysis of exhaled breath condensate in adults by nuclear magnetic resonance spectroscopy. Eur. Respir. J. 32, 1175–1183. 10.1183/09031936.00072408 18653649

[B10] De Laurentiis G. Paris D. Melck D. Montuschi P. Maniscalco M. Bianco A.. (2013). Separating smoking-related diseases using NMR-based metabolomics of exhaled breath condensate. J. Proteome Res. 12, 1502–1511. 10.1021/pr301171p 23360153

[B11] Djukanovic R. Sterk P. J. Fahy J. V. Hargreave F. E. (2002). Standardised methodology of sputum induction and processing. Eur. Respir. J. 37 (Suppl.), 1s−2s. 10.1183/09031936.02.00000102 12361359

[B12] Dweik R. A. Boggs P. B. Erzurum S. C. Irvin C. G. Leigh M. W. Lundberg J. O. (2011). American Thoracic Society Committee on interpretation of exhaled nitric oxide levels (F_E_NO) for clinical applications. An official ATS clinical practice guideline: interpretation of exhaled nitric oxide levels (FENO) for clinical applications. Am. J. Respir. Crit. Care Med. 184, 602–615. 10.1164/rccm.9120-11ST 21885636 PMC4408724

[B13] Effros R. M. Biller J. Foss B. Hoagland K. Dunning M. B. Castillo D.. (2003). A simple method for estimating respiratory solute dilution in exhaled breath condensates. Am. J. Respir. Crit. Care Med. 168, 1500–1505. 10.1164/rccm.200307-920OC 14512268

[B14] Efthimiadis A. Spanevello A. Hamid Q. Kelly M. M. Linden M. Louis R.. (2002). Methods of sputum processing for cell counts, immunocytochemistry and in situ hybridisation. Eur. Respir. J. 37 (Suppl.), 19s−23s. 10.1183/09031936.02.00001902 12361358

[B15] Fens N. de Nijs S. B. Peters S. Dekker T. Knobel H. H. Vink T. J.. (2011). Exhaled air molecular profiling in relation to inflammatory subtype and activity in COPD. Eur. Respir. J. 38, 1301–1309. 10.1183/09031936.00032911 21700610

[B16] Fens N. van Rossum A. G. Zanen P. van Ginneken B. van Klaveren R. J. Zwinderman A. H.. (2013). Subphenotypes of mild-to-moderate COPD by factor and cluster analysis of pulmonary function, CT imaging and breathomics in a population-based survey. COPD 10, 277–285. 10.3109/15412555.2012.744388 23536961

[B17] Fens N. Zwinderman A. H. van der Schee M. P. de Nijs S. B. Dijkers E. Roldaan A. C.. (2009). Exhaled breath profiling enables discrimination of chronic obstructive pulmonary disease and asthma. Am. J. Respir. Crit. Care Med. 180, 1076–1082. 10.1164/rccm.200906-0939OC 19713445

[B18] Global Initiative for Chronic Obstructive Lung Disease (GOLD) (2018). Global Strategy for The Diagnosis, Management, and Prevention of Chronic Obstructive Pulmonary Disease Available online at: www.goldcopd.org (Accessed Jan 8, 2018).

[B19] Hahsler M. Hornik K. Buchta C. (2008). Getting things in order: an introduction to the R package seriation. J. Stat. Soft. 25, 1–34. 10.18637/jss.v025.i03

[B20] Holden N. S. Rider C. F. Bell M. J. Velayudhan J. King E. M. Kaur M. (2010). Enhancement of inflammatory mediator release by beta_2_-adrenoceptoragonists in airway epithelial cells is reversed by glucocorticoid action. Br. J. Pharmacol. 160, 410–420. 10.1111/j.1476-5381.2010.00708.x 20423350 PMC2874862

[B21] Horvath I. Barnes P. J. Loukides S. Sterk P. J. Högman M. Olin A. C.. (2017). A European Respiratory Society technical standard: exhaled biomarkers in lung disease. Eur. Respir. J. 49:4. 10.1183/13993003.00965-2016 28446552

[B22] Lewis N. S. (2004). Comparisons between mammalian and artificial olfaction based on arrays of carbon black-polymer composite vapor detectors. Acc. Chem. Res. 37, 663–672. 10.1021/ar030120m 15379582

[B23] Lim S. H. Mix S. Anikst V. Budvytiene I. Eiden M. Churi Y.. (2016). Bacterial culture detection and identification in blood agar plates with an optoelectronic nose. Analyst 141, 918–925. 10.1039/c5an01990g 26753182

[B24] Montuschi P. Mondino C. Koch P. Barnes P. J. Ciabattoni G. (2006). Effects of a leukotriene receptor antagonist on exhaled leukotriene E_4_ and prostanoids in children with asthma. J. Aller. Clin. Immunol. 118, 347–353. 10.1016/j.jaci.2006.04.010 16890757

[B25] Montuschi P. Paris D. Melck D. Lucidi V. Ciabattoni G. Raia V.. (2012). NMR spectroscopy metabolomic profiling of exhaled breath condensate in patients with stable and unstable cystic fibrosis. Thorax 67, 222–228. 10.1136/thoraxjnl-2011-200072 22106016

[B26] Montuschi P. Ragazzoni E. Valente S. Corbo G. Mondino C. Ciappi G. (2003). Validation of 8-isoprostane and prostaglandin E_2_ measurements in exhaled breath condensate. Inflamm. Res. 52, 502–507. 10.1007/s00011-003-1212-6 14991078

[B27] Montuschi P. Santonico M. Mondino C. Pennazza G. Mantini G. Martinelli E.. (2010). Diagnostic performance of an electronic nose, fractional exhaled nitric oxide, and lung function testing in asthma. Chest 137, 790–796. 10.1378/chest.09-1836 20081096

[B28] Motta A. Paris D. Melck D. De Laurentiis G. Maniscalco M. Sofia M.. (2012). Nuclear magnetic resonance-based metabolomics of exhaled breath condensate: methodological aspects. Eur. Respir. J. 39, 498–500. 10.1183/09031936.00036411 22298616

[B29] Paggiaro P. Chanez P. Holz O. Ind P. W. Djukanovic R. Maestrelli P.. (2002). Sputum induction. Eur. Respir. J. 37(Suppl.), 3s−8s. 10.1183/09031936.02.00000302 12361361

[B30] Palama T. L. Canard I. Rautureau G. J. Mirande C. Chatellier S. Elena-Herrmann B. (2016). Identification of bacterial species by untargeted NMR spectroscopy of exo-metabolome. Analyst 141, 4558–4561. 10.1039/C6AN00393A 27349704

[B31] Revelle W. (2017). An Overview of the Psych Package. Package “psych” (version 1.6.9). Available online at: “https://cran.r-project.org/web/packages/psych/vignettes/overview.pdf” (Accessed: 9 January 2018).

[B32] Rochfort S. (2005). Metabolomics reviewed: a new “omics” platform technology for systems biology and implications for natural products research. J. Nat. Prod. 68, 1813–1820. 10.1021/np050255w 16378385

[B33] Röck F. Barsan N. Weimar U. (2008). Electronic nose: current status and future trends. Chem. Rev. 108, 705–711. 10.1021/cr068121q 18205411

[B34] Santini G. Mores N. Shohreh R. Valente S. Dabrowska M. Trové A.. (2016). Exhaled and non-exhaled non-invasive markers for assessment of respiratory inflammation in patients with stable COPD and healthy smokers. J. Breath. Res. 10:017102. 10.1088/1752-7155/10/1/017102 26814886

[B35] van der Schee M. P. Palmay R. Cowan J. O. Taylor D. R. (2013). Predicting steroid responsiveness in patients with asthma using exhaled breath profiling. Predicting steroid responsiveness in patients with asthma using exhaled breath profiling. Clin. Exp. Allergy 43, 1217–1225. 10.1111/cea.12147 24152154

[B36] van Velzen E. J. Westerhuis J. A. van Duynhoven J. P. van Dorsten F. A. Hoefsloot H. C. Jacobs D. M.. (2008). Multilevel data analysis of a crossover designed human nutritional intervention study. J. Proteome Res. 7, 4483–4491. 10.1021/pr800145 18754629

[B37] Wang Z. Ciabattoni G. Créminon C. Lawson J. Fitzgerald G. A. Patrono C. (1995). Immunological characterization of urinary 8-epi-prostaglandin F_2α_ excretion in man. J. Pharmacol. Exp. Ther. 275, 94–100. 7562601

[B38] Warnes G. R. Bolker B. Bonebakker L. Gentleman R. Liaw W. H. A. Lumley T. (2016). Various R Programming Tools for Plotting Data. Package “gplots” (version 3.0.1). Available online at: https://cran.r-project.org/web/packages/gplots/gplots.pdf (Accessed Jan 9, 2018).

[B39] Westerhuis J. A. van Velzen E. J. Hoefsloot H. C. Smilde A. K. (2010). Multivariate paired data analysis: multilevel PLSDA versus OPLSDA. Metabolomics 6, 119–128. 10.1007/s11306-009-0185-z 20339442 PMC2834771

[B40] Wilson A. D. Baietto M. (2009). Applications and advances in electronic-nose technologies. Sensors (Basel) 9, 5099–5148. 10.3390/s90705099 22346690 PMC3274163

