# Supplementary material for: Breathomics for Assessing the Effects of Treatment and Withdrawal With Inhaled Beclomethasone/Formoterol in Patients With COPD
Source: Front Pharmacol. 2018 Apr 17;9:258. doi: 10.3389/fphar.2018.00258 (PMC5914154; doi:10.3389/fphar.2018.00258)
Supplement: Supplementary file 1 [file Table1.docx]

**Table S1.** Classification accuracies and P values among different pharmacological treatments from visit 1 to visit 4 based on a monodimensional PLS model built on EBC NMR spectroscopy data in 14 patients with COPD. The significant variables in the univariate analysis are also reported (Wilcoxon signed-rank test P values).

| **Comparison** | **Overall accuracy** | **Variable (P value)** |
| --- | --- | --- |
| Visit 1 vs. Visit 2 | 42.0% (P > 0.05) | - |
| Visit 1 vs. Visit 3 | 55.3% (P > 0.05) | - |
| Visit 1 vs. Visit 4 | 72.0% (0.01) | Formate (0.029) |
| Visit 2 vs. Visit 3 | 53.3% (P > 0.05) | - |
| Visit 2 vs. Visit 4 | 63.3% (P > 0.05) | - |
| Visit 3 vs. Visit 4 | 57.3% (P > 0.05) | Acetate (0.009) |

Abbreviations: EBC, exhaled breath condensate; NMR, nuclear magnetic resonance; PLS, partial least squares.
