# Supplementary material for: Breathomics for Assessing the Effects of Treatment and Withdrawal With Inhaled Beclomethasone/Formoterol in Patients With COPD
Source: Front Pharmacol. 2018 Apr 17;9:258. doi: 10.3389/fphar.2018.00258 (PMC5914154; doi:10.3389/fphar.2018.00258)
Supplement: Supplementary file 2 [file Table2.docx]

**Table S2.** F_E_NO values in 14 patients with COPD at visit 1 to visit 4.*

|  | V1  (n = 14) | V2  (n = 14) | V3  (n = 14) | V4  (n = 14) | Overall P value |
| --- | --- | --- | --- | --- | --- |
| F_E_NO (ppb) | 21.9 (12.7-28) | 17.6 (12.3-20.3) | 18.5 (11.8-29) | 13.2 (11.8-21.7) | 0.35 |

*Data are expressed as medians and interquartile ranges. Within-group comparisons were performed with Friedman test and Wilcoxon signed rank test. Abbreviations: F_E_NO, fraction of exhaled nitric oxide; V, visit.
