# Supplementary material for: Breathomics for Assessing the Effects of Treatment and Withdrawal With Inhaled Beclomethasone/Formoterol in Patients With COPD
Source: Front Pharmacol. 2018 Apr 17;9:258. doi: 10.3389/fphar.2018.00258 (PMC5914154; doi:10.3389/fphar.2018.00258)
Supplement: Supplementary file 3 [file Table3.docx]

**Table S3.** PGE_2_ and 15-F_2t_-isoprostane concentrations in sputum and EBC*.

|  | V1 | V2 | V3 | V4 | Overall P value |
| --- | --- | --- | --- | --- | --- |
| n | 13 | 13 | 13 | 13 |  |
| Sputum PGE_2_  (pg/ml) | 18.6 (2-24.6)^1^ | 3.8 (2-54.9) | 40.1 (8.3-47)^1,2^ | 10.4 (3.5-20.8)^2^ | 0.04 |
|  | V1 | V2 | V3 | V4 | Overall P value |
| n | 9 | 9 | 9 | 9 |  |
| Sputum  15-F_2t_-isoprostane (pg/ml) | 25 (9.9-45.5) | 25.5 (13.2-45.4) | 33.2 (7.7-41.5) | 13.5 (7.8-26.2) | 0.84 |
|  | V1 | V2 | V3 | V4 | Overall P value |
| n | 12 | 12 | 12 | 12 |  |
| EBC PGE_2_  (pg/ml) | 18.7 (10.6-30.4) | 19.1 (11.9-55.1) | 14.7 (7-26.1) | 11.3 (4-18.7) | 0.19 |
|  | V1 | V2 | V3 | V4 | Overall P value |
| n | 14 | 14 | 14 | 14 |  |
| EBC  15-F_2t_-isoprostane (pg/ml) | 18.3 (1.4-37) | 24 (9.7-35.7) | 20.8 (15.1-32.8) | 21.3 (8.1-32.7) | 0.60 |

*Data are expressed as median and interquartile range. Within-group, between-visit comparisons were performed with Friedman’s test. If overall P was lower than 0.05, considered significant, Wilcoxon signed rank test was performed.

^1^: P = 0.021; ^2^: P = 0.008.

One patient with COPD had undetectable sputum PGE_2_ concentrations at all visits (4 samples). Five patients with COPD had undetectable sputum 15-F_2t_-isoprostane concentrations at all visits (20 samples). Two patients with COPD had undetectable EBC PGE_2_ concentrations at all visits (8 samples). At undetectable samples, an arbitrary concentration value of 1 pg/ml, corresponding to 50% of the analytical technique detection limit (2 pg/ml), was assigned.

Abbreviations: EBC, exhaled breath condensate; PGE_2_, prostaglandin E_2_; V, visit.
