# Supplementary material for: Breathomics for Assessing the Effects of Treatment and Withdrawal With Inhaled Beclomethasone/Formoterol in Patients With COPD
Source: Front Pharmacol. 2018 Apr 17;9:258. doi: 10.3389/fphar.2018.00258 (PMC5914154; doi:10.3389/fphar.2018.00258)
Supplement: Supplementary file 4 [file Table4.docx]

**Table S4.** Within-group comparison of percentage sputum cell counts in the 8 patients with COPD who had a complete set of sputum slides (visit 1 to visit 4)*.

|  | V1 | V2 | V3 | V4 | P value |
| --- | --- | --- | --- | --- | --- |
| n | 8 | 8 | 8 | 8 |  |
| Neutrophils, % | 82.5 (57.3-91.6) | 85.5 (77.6-95.5) | 86.5 (72.3-93.9) | 83.3 (68.4-89.6) | 0.55 |
| Macrophages, % | 10.5 (4.1-39.8) | 11.3 (2.8-17.4) | 10 (1.7-15) | 7.8 (4.4-18) | 0.91 |
| Eosinophils, % | 0 (0-0.5) | 0 (0-0.8) | 0.5 (0-2.9) | 0.5 (0-3.3) | 0.20 |
| Lymphocytes, % | 0 (0-0.4) | 0 (0-0.5) | 0 (0-0.4) | 0 (0-0.4) | 0.87 |

*Data are expressed as medians and interquartile range. Percentage of basophil and bronchial epithelial cell counts was 0 and is not shown. Within-group comparisons were performed with Friedman test. Abbreviation: V, visit.
