# Supplementary material for: Breathomics for Assessing the Effects of Treatment and Withdrawal With Inhaled Beclomethasone/Formoterol in Patients With COPD
Source: Front Pharmacol. 2018 Apr 17;9:258. doi: 10.3389/fphar.2018.00258 (PMC5914154; doi:10.3389/fphar.2018.00258)
Supplement: Supplementary file 5 [file Table5.docx]

**Table S5.** Percentage sputum cell counts in all valid sputum slides (n = 43)*.

|  | V1 | V2 | V3 | V4 | P value |
| --- | --- | --- | --- | --- | --- |
| n | 10 | 12 | 11 | 10 |  |
| Neutrophils, % | 85.8 (67.8-93.4) | 87.5 (78-96) | 87 (73-89) | 83.5 (72.1-90.8) | n.s. |
| Macrophages, % | 10.5 (2.9-25.3) | 7.8 (1.8-17.4) | 11 (3-13.5) | 7.8 (4-18) | n.s. |
| Eosinophils, % | 0.3 (0-0.6) | 0 (0-1) | 0 (0-1) | 0.5 (0-2.8) | n.s. |
| Lymphocytes, % | 0 (0-0.1) | 0 (0-0.4) | 0 (0-0) | 0 (0-1) | n.s. |

*Data are expressed as medians and interquartile range. Thirteen sputum slides were excluded due to squamous cell counts > 30%. Sputum cell counts are expressed as a percentage of total non-squamous cells. Percentage of basophils and bronchial epithelial cells was 0. Comparisons were performed with Friedman test. Abbreviations: n.s., not significant for all comparisons within the group; V, visit.
