# Supplementary material for: Breathomics for Assessing the Effects of Treatment and Withdrawal With Inhaled Beclomethasone/Formoterol in Patients With COPD
Source: Front Pharmacol. 2018 Apr 17;9:258. doi: 10.3389/fphar.2018.00258 (PMC5914154; doi:10.3389/fphar.2018.00258)
Supplement: Supplementary file 6 [file Presentation1.pdf]

Online Supplementary Material

BREATHOMICS FOR ASSESSING THE EFFECTS OF TREATMENT AND WITHDRAWAL WITH INHALED BECLOMETHASONE/FORMOTEROL IN PATIENTS WITH COPD

[1]Paolo Montuschi, M.D., [1]Giuseppe Santini, B.Sci., [1]Nadia Mores, M.D., [2]Alessia Vignoli, M.Sci., [3]Francesco Macagno, M.D., [4]Rugia Shoreh, PhD, [2]Leonardo Tenori, PhD, [5]Gina Zini, M.D., [3]Leonello Fuso, M.D., [6]Chiara Mondino, M.D., [7]Corrado Di Natale, PhD, [7]Arnaldo D'Amico, PhD, [2]Claudio Luchinat, PhD, [8]Peter J. Barnes, DM, [9]Tim Higenbottam, M.D.

METHODS

Study subjects

Subjects were on maintenance treatment with inhaled fluticasone propionate/salmeterol at a constant dose of 500/50 μg b.i.d. delivered via a dry powder inhaler (DPI) for at least 8 weeks.

Patients were recruited at the outpatient clinic, Respiratory Medicine Unit, and Clinical Pharmacology Unit, Catholic University of the Sacred Heart, University Hospital Agostino Gemelli Foundation, Rome, Italy.

Study design

Treatment with an extrafine beclomethasone/formoterol fixed dose combination (FDC) was suspended and reintroduced as described in Figure 1 (main manuscript).

At visit 1, patient treatment, consisting of a constant dose of inhaled fluticasone propionate/salmeterol xynafoate FDC (500/50 μg b.i.d. via DPI) for at least 8 weeks, was switched to an extrafine formulation of inhaled beclomethasone/formoterol FDC (100/6 μg 2 puffs b.i.d. via

pressurized metered dose inhaler [pMDI]). After 2 week treatment (visit 2), FDC containing beclomethasone was suspended, while maintaining formoterol alone (6 μg 2 puffs b.i.d. via pMDI). After 4 weeks of beclomethasone withdrawal (visit 3), treatment with inhaled extrafine beclomethasone/formoterol FDC at the same dose (100/6 μg 2 puffs b.i.d. via pMDI) was reintroduced and maintained for 4 weeks (visit 4). Study duration was 10 weeks. Study design and details of intervention are shown in Figure 1 (main manuscript). At each visit, interventions were performed in the following order: fraction of exhaled nitric oxide (F_ENO) measurement, exhaled breath sampling for e-nose analysis, exhaled breath condensate (EBC) collection, pulmonary function tests, and sputum induction. Skin prick testing was performed at visit 1.

Before breath sampling, subjects were asked to refrain from eating and drinking (except water) for at least 12 h and to stop short-acting bronchodilators for at least 12 h and long-acting bronchodilators for at least 24 h. Breath sampling and e-nose analysis was performed in a windowless conference facility in the Clinical Pharmacology Unit, University Hospital Agostino Gemelli Foundation, Catholic University of the Sacred Heart, controlled by a central ventilation system and without disinfectant dispensers or person traffic.

Collection of exhaled breath

Exhaled breath was collected through mixed expiratory sampling in which total breath, including dead space air, is collected. An equilibration phase (wash-in) with volatile organic compound (VOC)-filtered room air was performed before breath sampling to reduce the interference of ambient VOCs (Fens et al, 2009; Bofan et al, 2013). Subjects were asked to breathe tidally VOC-filtered air for 5 minutes, while wearing a nose-clip, into a 2-way non-rebreathing valve with an inspiratory VOC filter and an expiratory silica reservoir to reduce sample water vapor as this could affect sensor response (Rock et al, 2008; Wilson and Baietto, 2009). Then, subjects were asked to inhale to maximal inspiration and perform a FVC manoeuvre into a Tedlar bag against an expiratory resistance of 20 cmH_2O to close the soft palate and obtain an expiratory flow of 0.1-0.2

L/sec (Fens et al, 2009; Bofan et al, 2013). This breath sampling procedure minimizes the effect of ambient VOCs on e-nose analysis.

Electronic nose analysis

The setup for e-nose analysis used in this study consists of a commercially available e-nose (Cyranose 320®, Sensigent, Baldwin Park, USA, formerly manufactured by Smiths Detection, Pasadena, USA) (Fens et al, 2009; Lewis, 2004) and a prototype e-nose (Ten 2011, University of Rome Tor Vergata, Italy) (Montuschi et al, 2010). The commercially available e-nose consists of an array of 32 chemical sensors made from composites of an inorganic conductor (carbon black) and insulating organic polymers (Lewis, 2004). The measurement is based on a resistance variation in each chemical sensor when exposed to breath VOCs. E-nose analysis was performed immediately after breath sample collection. Each breath sample was analyzed 5 times with the same e-nose. Data from the first measure were discarded as suggested by the manufacturer and reported in previous studies (Fens et al, 2009; Dragonieri et al, 2007); the second e-nose measure was used for data analysis (Bofan et al, 2013). E-nose responses for each sensor (changes in resistance) were stored in the e-nose in-built database, copied into a Matlab file and analyzed offline with a pattern recognition algorithm (Bofan et al, 2013). Algorithms were created using principle component analysis (PCA) and multilevel partial least square (PLS) analysis. The K-nearest neighbours method, applied on the multilevel PLS scores, was used for classification purposes. Only the first few principal components were used to avoid errors of the first degree. Component number was chosen based on those required to describe 99.9% of the dataset variance. T-test was used to identify discriminating principle components, with a $p < 0.1$ considered significant, which were then analyzed with canonical discriminant analysis. The Monte Carlo cross-validation scheme was used to calculate the cross-validated sensitivity, specificity and global classification accuracy of the algorithm as the limited sample size precluded training and testing validation. The analysis was performed with SPSS (version 17.0, SPSS Inc., Chicago, IL, USA).

The second e-nose, which detects frequency variations, contains an array of eight quartz microbalance (QMB) gas sensors coated by molecular films of metallo-porphyrins (Montuschi et al, 2010). Sensors detect the amount of molecules absorbed in a sensitive film through the changes of resonant frequency that is proportional to the absorbed mass (Montuschi et al, 2010).

EBC sampling

The condenser has a saliva trap to reduce the chance of salivary contamination Montuschi et al, 2012). Under our experimental conditions, no effect of salivary contamination or cleaning solution on EBC profiles has been observed (Montuschi et al, 2012; De Laurentiis et al, 2008, Motta et al, 2012). Subjects were asked to wash their mouth thoroughly before collecting EBC, to breathe tidally through a mouthpiece into a two-way non-rebreathing valve for 15 minutes wearing a nose-clip, and to stop breathing into the mouthpiece and swallow every time they felt salivation. An average of 1.5 ± 0.2 ml (mean ± SD) of EBC was collected in 15 minutes of tidal breathing. EBC sampling was performed as previously described (Montuschi et al, 2012). EBC was immediately transferred into 10 ml glass vials, closed with 20 mm butyl rubber lined with polytetrafluoroethylene septa and crimped with perforated aluminium seals (Montuschi et al, 2012) . Before sealing, volatile substances were removed from samples by a gentle nitrogen gas flow for 3 min (Montuschi et al, 2012).[Previous experiments showed no difference with spectra acquired after a variable time of nitrogen exposure (1, 3, 5, 10, 15 and 20 min).[9] However, as 1-min interval appeared to be too short to avoid systematic errors, a 3-min interval was chosen. Samples were not dried out to avoid their precipitation, with a possible loss of nonvolatile compounds, and/or formation of aggregates upon dissolving the dried condensate for NMR measurements. Sealed samples were then frozen in liquid nitrogen, so as to immediately "quench" metabolism and preserve the metabolite concentrations (Montuschi et al, 2012; De Laurentiis et al, 2008). Samples were stored at -80°C until metabolomic analysis.

Metabolomic analysis of EBC with NMR spectroscopy

EBC samples, stored at -80°C, were thawed at room temperature. Sample aliquots (630 μL) were then centrifuged at 14000 rpm for 10 minutes. Sample aliquots of the supernatant (540 μL) were mixed with 55 μL of $^{2}H_2O$ and 5 μL of buffer phosphate (0.2 M Na_2HPO_4, 0.2 M NaH_2PO_4 in 100% $^{2}H_2O$, pH 7) containing 30 mM NaN_3 and 10 mM sodium 3-trimethylsilyl [2,2,3,3-2H4] propionate (TMSP). Then, 450 μL of mixture were pipetted into 4.25 mm NMR tube (Bruker BioSpin) (Bertini et al, 2014).

^{1}H-NMR spectra were acquired using a Bruker 600 MHz spectrometer (Bruker BioSpin) operating at 600.13 MHz proton Larmor frequency and equipped with a 5 mm CPTCI ^{1}H-^{13}C/^{31}P-^{2}H cryo-probe including a z-axis gradient coil, an automatic tuning-matching (ATM), and an automatic sample changer (Bertini et al, 2014).

A PT 100 thermocouple served for temperature stabilization at the level of approximately 0.1 K at the sample. Before measurement, samples were kept for at least 3 min inside the NMR probe head for temperature equilibration (300 K). For each sample, a one-dimensional NMR spectrum was acquired with water peak suppression using a standard pulse sequence (NOESY presat), using 512 free induction decays (FIDs), 64 k data points, a spectral width of 12019 Hz, an acquisition time of 2.7 s, a relaxation delay of 4 s, and a mixing time of 100 ms (Bertini et al, 2014).

Free induction decays were multiplied by an exponential function equivalent to 1.0 Hz line-broadening factor before applying Fourier transform. Transformed spectra were manually corrected for phase and baseline distortions, and calibrated (TMSP peak at 0.00 ppm) using TopSpin (Bruker). Each one-dimensional spectrum in the range of 0.02–10 ppm was segmented into 0.02-ppm chemical shift buckets, and the corresponding spectral areas, after scaling, were used as input variables for the subsequent statistical analysis (Bertini et al, 2014). Regions

between 6.0 and 4.5 ppm, containing residual water signals, were removed. Probabilistic quotient normalization (PQN) algorithm (Dieterle et al, 2006) was chosen to normalize the spectra, because it is a more accurate and robust alternative to the total area scaling, and PQN performs well even when large variations of dilution occur. Signals were assigned on template one dimensional NMR profiles by using matching routines of AMIX 3.8.4 (Bruker BioSpin) in combination with the BIOREFCODE reference database (Bruker BioSpin), and publicly available databases like HMDB (Human Metabolome DataBase) (Wishart et al, 2007).

All data analysis were performed using R, an open source software for the analysis of statistical data (Ihaka and Gentleman, 1996). Multivariate data analyses were conducted on processed data by combining established methods. Data reduction was obtained by means of multilevel Partial Least Square Analysis (mPLS) using the algorithm implemented in the R-library "plsgenomics" and the standard R function "cancor".

For the purpose of classification, we used the K-nearest neighbors (k-NN) method (k = 3) applied on the multilevel PLS scores (Cover and Hart, 1967). All the accuracies reported and the confusion matrix for the different classifications were assessed by means of 100 cycles of a Monte Carlo cross-validation scheme (MCCV, R script in-house developed) (Bijlsma S et al, 2006; Boulesteix, 2015). Briefly, 90% of the data were randomly chosen at each iteration as a training set to build the model. Then the remaining 10% was tested and sensitivity, specificity and accuracy for the classification were assessed.

For the univariate analysis, after PQN normalization of the spectra, each spectral region related to the metabolites assigned in the ^{1}H NMR profiles was aligned, by a simple horizontal rigid shift, in a way that the corresponding peaks of interest resulted superimposed. Then, they were integrated reducing the selected ppm intervals, and improving the precision in the integration by compensating the variations in peaks position in the different EBC samples. The obtained integrals represent the metabolite concentrations in arbitrary units. The whole procedure was performed using a R script developed in house. EBC metabolite concentrations at each visit

were determined. Statistical significance was assessed using the univariate non-parametric Wilcoxon signed-rank test (Wilcoxon, 1945). A p-value < 0.05 was deemed statistically significant.

To evaluate the discrimination power of the integrated model containing all the different analytical approaches used in this study, paired visit comparisons were performed using data matrices created by binding together all the different variables obtained from the various techniques (EBC NMR-spectroscopy, spirometry, carbon polymer sensor e-nose, quartz crystal sensor e-nose, eicosanoids and F_ENO measurements, sputum cells). On this dataset, the same multivariate statistical approach previously described was applied (multilevel PLS and cross-validation using k-NN). Furthermore, the accuracies calculated were assessed for significance against the null hypothesis of no prediction accuracy in the data by means of 10^2 randomized class-permutations test (Alonso et al, 2015).

Measurement of PGE_2 and 15-F_{2t}-isoprostane in sputum supernatants and EBC

PGE_2 and 15-F_{2t}-isoprostane concentrations in sputum supernatants and EBC were measured with radioimmunoassays developed in our laboratory that were previously validated and compared with gas chromatography/mass spectrometry (GC/MS) and high performance liquid chromatography (HPLC) (Wang et al, 1995; Montuschi et al, 2003).

Sputum cell analysis

Sputum induction, processing and analysis were performed according to the European Respiratory Society (ERS) guidelines (Djukanovic et al, 2002; Paggiaro et al, 2002). Baseline FEV_1 was recorded before sputum induction. Subjects were pre-treated with inhaled salbutamol (400 µg), and, after 10 min, a spirometry was repeated. (Paggiaro et al, 2002) Subjects were asked to inhale hypertonic saline (3%) for 5 min and, then, to rinse their mouths and try to expectorate into a sterilized box (Paggiaro et al, 2002). Five-minute inhalation sessions were repeated four times for a

total of 20 min (Paggiaro et al, 2002). A spirometry was performed after each inhalation session to detect significant fall of FEV_1. The procedure was stopped when approximately 1 g of plugs was collected, or if patients had symptoms or/and if FEV_1 was reduced more than 20% over baseline values (Paggiaro et al, 2002). Sputum was processed within 2 h to ensure optimum cell counting and staining, with the sample always kept in ice (Efthimiadis et al, 2002). 100-500 mg sputum was selected for sputum analysis. Dithiothreitol (DTT) 0.1% was added to sputum samples which were kept in a shaking rocker at room temperature for 20 minutes for sample homogenization (Efthimiadis et al, 2002). Samples were filtered through a 48 μm nylon mesh into a pre-weighed conical tube and filtrate was weighed. Total cell count was performed manually using a haemocytometer and cell viability was assessed by the trypan blue exclusion method before centrifugation (Efthimiadis et al, 2002). To separate cell pellet from sputum supernatants, samples were centrifuged at 4°C for 10 min with a centrifugal force of 1200 x g (Efthimiadis et al, 2002). Sputum supernatant samples were collected and stored at −80°C for measurement of PGE_2 and 15-F_{2t}-isoprostane concentrations. Cell pellets were resuspended in PBS buffer and cell concentrations were adjusted to 1 x 10^6 cells/ml. Cytospins were prepared by adding 40-60 μl of cell resuspension to each cytospin and using a Shandon cytocentrifuge at 22 x g for 6 min (Efthimiadis et al, 2002). Cytospins were stained for differential cell counts using Giemsa staining (Efthimiadis et al, 2002). The differential cell counts was performed by counting a minimum of 400 nonsquamous cells and reported as the relative numbers of eosinophils, neutrophils, macrophages, lymphocytes, and bronchial epithelial cells, expressed as a percentage of total nonsquamous cells (Efthimiadis et al, 2002). The percentage of squamous cells was reported separately. Slides with squamous cells > 30% of total cells were discarded. Slides were read blindly by two qualified and fully trained physicians. Monthly quality control was performed including internal slide reading and equipment calibration (Efthimiadis et al, 2002).

Multivariate data analysis

To investigate the correlations among the different analytical approaches used in this study, a matrix with data from all techniques was created and then its correlation matrix was calculated using the algorithm implemented in the R-library "psych" (Hahsler et al, 2008; Revelle, 2016). The heatmap of the correlation matrix was built using the R-function "heatmap.2", implemented in the "gplots" package (Warnes et al, 2016). This function reordered the rows and columns of the correlation matrix according to the restrictions imposed by the dendrogram, calculated on the basis of the Euclidean distance among the data and using the R-function "hclust".

Statistical analysis

Data were expressed as mean ± SEM or medians and interquartile ranges (25th and 75th percentiles), after assessing for normality with the D'Agostino-Pearson omnibus normality test. Depending on data distribution, repeated-measures ANOVA or Friedman test were used for assessing within-group pharmacological treatment effect. If overall statistical significance was observed, unpaired t test and Mann-Whitney U test were used for comparing groups for normally distributed and nonparametric data, respectively. Correlation was expressed as a Pearson coefficient. Significance was defined as a value of $p < 0.05$.

RESULTS

Study subjects

The mean age of the subjects (2 females and 12 males) enrolled in this study was 73.6 ± 1.8 years. Patients smoke a mean of 64.3 ± 1.8 pack-year and, according to the airflow limitation severity, were classified as GOLD I (5 patients), II (5 patients), and III (4 patients). Information about common aeroallergens, history of atopy, sputum eosinophils at visit 1 are detailed in Table 1.

Pulmonary function testing

All patients with COPD performed spirometry at all visits. There was no missing data.

An Excel table with lung function test values for individual patients with COPD is provided as online supplementary material. Results of pulmonary function tests show higher mean absolute and percentage of predicted pre-bronchodilator $FEF_{25\%-75\%}$ values after treatment with inhaled beclomethasone/formoterol FDC in microparticle formulation (visit 2) compared with inhaled formoterol alone in microparticle formulation (visit 3) (Figure 2A and 2B). These data suggest that inhaled ultrafine beclomethasone/formoterol FDC improves small airway function as reflected by $FEF_{25\%-75\%}$ values. This might be particularly relevant as this functional effect is observed after only 4 week treatment with inhaled beclomethasone/formoterol, a relatively short duration of treatment for COPD trials, and in view of the fact that all patients with COPD included in our study had normal sputum eosinophils, negative reversibility test to bronchodilators, negative skin prick tests, and no history of atopy, thus, excluding an asthma component, on which ICS are generally more effective.

An effect of ICS on small airway function is also suggested by a higher mean pre-bronchodilator FEV_1/FVC ratio at visit 1 (COPD patients were on inhaled fluticasone/salmeterol FDC at full doses) compared with visit 3 (post-treatment with inhaled formoterol alone) (Table 2).

Higher mean absolute and percentage of pre-bronchodilator PEF predicted values were observed after 4 week treatment with inhaled beclomethasone/formoterol (visit 2) compared with visit 1 (Figure 2C and 2D, Table 2). These data might be the consequence of a higher lung deposition of the ultrafine drug formulation delivered through pMDI, resulting in a greater anti-inflammatory and/or bronchodilating effect of inhaled beclomethasone/formoterol combination in the small airways compared with inhaled fluticasone/salmeterol in standard formulation delivered through DPI (Table 2). No within-group differences in post-bronchodilator functional parameters were observed (Table 3).

Electronic nose

E-nose analysis with carbon polymer sensor e-nose and quartz crystal sensor e-nose was performed in all 14 subjects at all 4 visits for a total of 56 breathprints for each e-nose. Results are shown in Table 4 and 5. Data were normally distributed and expressed as mean ± SEM. Mean values (n = 14) for each single sensor at each visit (V1-V4) are shown. Within- group differences were assessed by ANOVA for repeated measures and paired t test using GraphPad Prism software.

Regarding analysis with carbon polymer sensor e-nose, mean ΔR values in 15 out of 32 sensors showed significant differences between visit 1 (screening visit) and visit 4 (post-treatment visit) (sensor 1, 2, 4, 7, 8, 12, 14, 15, 16, 19, 21, 24, 25, 27, 32) and mean ΔR values in 7 sensors showed significant differences between visit 1 and visit 2 (sensor 2, 8, 19, 21, 24, 25, 27).

As all patients with COPD were on a constant dose of a FDC of inhaled fluticasone/salmeterol 500/50 μg one puff b.i.d. via DPI (Seretide® or Aliflus Diskus®) for at least 8 weeks before study enrolment, these data indicate that the FDC of inhaled beclomethasone/formoterol (Foster®) via pMDI at a dose of two puffs of 100/6 μg b.i.d. has a different effect on carbon polymer sensor e-nose breathprints. However, the pathophysiological and clinical implications of these differences are currently unknown and require further study.

There was no difference in the relative change in resistance [(Rmax-R0)/R0] of any sensor between visit 2 (baseline visit) and visit 3 (post-withdrawal visit). These data suggest a similar effect of inhaled ultrafine beclomethasone/formoterol and inhaled ultrafine formoterol alone on e-nose breathprints in patients with mild to severe COPD. This evidence is reinforced by similar results obtained with the quartz crystal sensor e-nose, based on a different technology (see below).

There was no difference in the behaviour of all sensors between visit 2 (baseline visit) and visit 4 (post-treatment visit).

Consistent with carbon polymer sensor behaviour, two quartz crystal sensors showed significant differences between visit 1 and visit 4, whereas between-visit difference in sensor 3 (P = 0.056) and

5 (P = 0.059) response was close to statistical significance. Sensor 3 and 4 showed significant differences between visit 1 and visit 2, whereas between-visit differences in sensor 2 (P = 0.054) and 5 (P = 0.093) approached statistical significance (Table 5).

These results are consistent with and confirm those obtained with the carbon polymer sensor array, an e-nose based on a different technology (see above), indicating a robust methodology.

Metabolomic analysis of EBC with NMR spectroscopy

EBC samples obtained from 14 patients with COPD at visit 1 to visit 4 for a total of 56 EBC samples were analyzed with NMR spectroscopy as described in Methods. There was no missing data.

When EBC spectra were analyzed with multilevel PLS, that is analysis of pairs of spectra from the same subject at two different visits, comparison between visit 1 and visit 4 showed a classification accuracy of 72% (Supplementary Figure 2).

These data indicate that regular treatment with inhaled fluticasone/salmeterol (COPD patients were on this FDC at a constant full dose, delivered through DPI, for at least 8 weeks) is associated with a different EBC metabolite profile compared with that observed after 4-week treatment with inhaled beclomethasone/formoterol in microparticle formulation delivered through pMDI. The pathophysiological meaning and possible implications of these results require further research.

Regarding the other paired comparisons, classification accuracy was below 70% (s-Table 1). The lack of between visit differences in EBC metabolite profiles, apart from visit 1 vs visit 4 (formate) and visit 3 vs visit 4 (acetate), could be due to high intragroup inter-individual variability in EBC metabolites which might mask pharmacological treatment-induced differences.

Among metabolites responsible for discrimination between visit 1 and visit 4, formate was the only one with a P-value < 0.05. EBC formate levels were lower at visit 4 (409.16 ± 224.76, relative intensity) compared with those observed at visit 1 (694.95 ± 360.47, relative intensity; P = 0.029) (Supplementary Figure 3A and 3B). EBC formate levels were also lower at visit 4 (409.16 ±

224.76, relative intensity) compared with those observed at visit 2 (695.14 ± 519.50, relative intensity; P = 0.049) (Table 6), although accuracy of classification between visit 2 and visit 4 based on EBC NMR spectroscopy did not reach the significant threshold of 0.7 (0.63) (s-Table 1). Moreover, at visit 4 EBC acetate levels were lower than those measured at visit 3 (P = 0.01) (s-Table 3). The pathophysiological meaning and possible implications of these findings require further research.

Measurement of fraction of exhaled nitric oxide (F_ENO)

Measurement of F_ENO was performed in all subjects at each visit. There was no missing data. There was no within-group difference in F_ENO concentrations in patients with COPD (overall P = 0.3529) (s-Table 2).

However, in the 8 patients with COPD who had elevated F_ENO concentrations (> 20 ppb), median F_ENO was lower after treatment with beclomethasone/formoterol FDC (visit 4) than after treatment with formoterol alone (visit 3) (26.6 (12.8-34.) ppb vs 14.9 (13.1-25.7) ppb), although this difference was not statistically significant (P = 0.23).

In the same group of patients with COPD and elevated F_ENO, the difference in median F_ENO concentrations after treatment with fluticasone/salmeterol FDC (visit 1) (27.2 (23.2-29.3) ppb) and after 2-week treatment with beclomethasone/formoterol FDC (visit 2) (19.7 (12.8-34.7) ppb) was very close to statistical significance (P = 0.058, Wilcoxon matched-pairs signed rank test).

Taken together, this evidence might suggest a selective effect of inhaled extrafine beclomethasone on F_ENO in those patients with COPD who have higher F_ENO concentrations at baseline. As patients with COPD at visit 1 were on a constant full dose of inhaled fluticasone, delivered through DPI, for at least 2 months, further reduction in F_ENO concentrations after 2-week treatment with inhaled beclomethasone (visit 2), might reflect a greater anti-inflammatory effect of the microparticle formulation.

Measurement of PGE_2 in sputum supernatants

PGE_2 concentrations were detected in 49 out of a total of 56 sputum samples. In 7 samples, sputum PGE_2 concentrations were undetectable. One patient with COPD had undetectable sputum PGE_2 concentrations at all visits (4 samples) and was excluded from statistical analysis. At the other 3 undetectable samples, an arbitrary concentration value of 1 pg/ml, corresponding to 50% of the analytical technique detection limit (2 pg/ml), was assigned.

Median sputum PGE_2 concentrations in patients with COPD at visit 1 to visit 4 are shown in s-Table 3.

Compared with visit 3, lower sputum PGE_2 concentrations were observed at visit 4 ($P = 0.008$) and at visit 1 ($P = 0.021$) (s-Table 3).

Median sputum PGE_2 concentrations at visit 2 (post-treatment with inhaled beclomethasone/formoterol) were lower (3.75 (1.98-54.9) pg/ml) than those observed at visit 3 (post-treatment with inhaled formoterol alone) (40.1 (8.3-47.0) pg/ml), although this difference was not statistically significant ($P = 0.21$), likely due to the high inter-individual variability.

These data suggest that treatment with both ICS combinations, containing either fluticasone propionate or beclomethasone, reduce sputum concentrations of PGE_2, an eicosanoid which has pro-inflammatory effects in the airways.

Measurement of 15-F_{2t}-isoprostane in sputum supernatants

15-F_{2t}-isoprostane concentrations were detected in 30 out of a total of 56 sputum samples. In 26 samples, sputum 15-F_{2t}-isoprostane concentrations were undetectable. Five patients with COPD had undetectable sputum 15-F_{2t}-isoprostane concentrations at all visits (20 samples) and were excluded from statistical analysis. At the other 6 undetectable samples, an arbitrary concentration value of 1 pg/ml, corresponding to 50% of the analytical technique detection limit (2 pg/ml), was assigned.

Median sputum 15-F_{2t}-isoprostane concentrations in patients with COPD at visit 1 to visit 4 are shown in s-Table 3.

There was no within-group difference in sputum 15-F_{2t}-isoprostane concentrations (P = 0.84) (s-Table 3) suggesting that this biomarker of oxidative stress is resistant to ICS treatment in line with previous studies (Montuschi et al, 2000).

Measurement of PGE_2 in EBC

PGE_2 concentrations were detected in 43 out of a total of 56 EBC samples. In 13 samples, EBC PGE_2 concentrations were undetectable. Two patients with COPD had undetectable EBC concentrations at all visits (8 samples) and were excluded from statistical analysis. At the other 5 undetectable samples, an arbitrary concentration value of 1 pg/ml, corresponding to 50% of the analytical technique detection limit (2 pg/ml), was assigned.

Median EBC PGE_2 concentrations in patients with COPD at visit 1 to visit 4 are shown in s-Table 3.

There was no within-group difference in EBC PGE_2 concentrations (P = 0.19) (s-Table 3).

Measurement of 15-F_{2t}-isoprostane in EBC

15-F_{2t}-isoprostane concentrations were detected in 46 out of a total 56 EBC samples. In 10 samples, EBC 15-F_{2t}-isoprostane concentrations were undetectable. At these samples, an arbitrary concentration value of 1 pg/ml, corresponding to 50% of the analytical technique detection limit (2 pg/ml), was assigned.

Median EBC 15-F_{2t}-isoprostane concentrations in patients with COPD at visit 1 to visit 4 are shown in s-Table 3.

There was no within-group difference in EBC 15-F_{2t}-isoprostane concentrations (P = 0.60) (s-Table 3) suggesting that this biomarker of oxidative stress is resistant to ICS treatment in line with previous studies (Montuschi et al, 2000).

Sputum cell analysis

Sputum was successfully induced and collected in all patients at all visits. Fifty-six sputum samples were collected. Corresponding cytospins were prepared and slides were stained with May-Grunwald-Giemsa as described Methods. Sputum slides were read as described in Methods. Thirteen sputum slides were discarded because of high salivary squamous cells (> 30% of total sputum cells). No patient with COPD had sputum eosinophilia, as defined by sputum cell counts > 3%, at visit 1 (screening visit).

Medians and interquartile range of sputum cell types in the 8 patients with COPD who had a complete set of sputum slides are shown in s-Table 4. Neutrophils were the predominant cell types in sputum obtained from patients with COPD.

Within-group comparison was performed with Friedman test in the eight patients who had a complete set of sputum slides (visit 1 to visit 4).

There was no within-group difference in neutrophil, macrophage, eosinophil, basophil, bronchial epithelial cell counts (s-Table 5). Basophil and bronchial epithelial cell counts are not shown in Table 5 as counts were mostly 0.

Correlations

Correlations between multidimensional variables in 14 patients with COPD at visit 1 to visit 4 (n = 56) are shown as a heatmap (Figure 4).

There was a negative correlation between quartz crystal sensor 4 (r = -0.35, P = 0.023) and 7 (r = -0.31, P = 0.041) and sputum neutrophil cell counts (Figure 4). Negative correlations between quartz crystal sensor 3 (r = -0.26, P = 0.088), 5 (r = -0.29, P = 0.056) and 6 (r = -0.27, P = 0.075) and sputum neutrophils were close to statistical significance.

Apart from carbon polymer sensor 2 (r = 0.31, P = 0.021), 3 (r = 0.30, P = 0.026), 4 (r = 0.35, P = 0.007), 5 (r = 0.51, P = 6.58^{-5}), 6 (r = 0.27, P = 0.048), 9 (r = 0.31, P = 0.019), 12 (r = 0.28, P =

0.037), 15 ($r = 0.31$, $P = 0.022$), 18 ($r = 0.27$, $P = 0.042$), 19 ($r = 0.29$, $P = 0.031$), 23 ($r = 0.45$, $P = 0.001$), 25 ($r = 0.31$, $P = 0.022$), 26 ($r = 0.35$, $P = 0.007$), 28 ($r = 0.29$, $P = 0.028$), 29 ($r = 0.32$, $P = 0.016$), 30 ($r = 0.32$, $P = 0.015$), 31 ($r = 0.51$, $P = 6.89^{-5}$), which were correlated with EBC phenol concentrations, there was no correlation between carbon polymer sensors and other outcome measures, including pulmonary function tests, sputum neutrophil and macrophages cell counts, PGE_2 concentrations in sputum supernatants and EBC, and 15-F_{2t}-isoprostane concentrations in sputum supernatants and EBC (Figure 4).

FEV_1/FVC values were negatively correlated with sputum neutrophil cell counts ($r = -0.34$; $P = 0.028$) (Figure 4). Negative correlations between sputum neutrophils and FEV_1 percentage of predicted values ($r = -0.30$, $P = 0.053$) and $FEF_{25\%-75\%}$ percentage of predicted values ($r = -027$, $P = 0.075$) were close to statistical significance (Figure 4).

There was a correlation between EBC PGE_2 and formate ($r = 0.78$, $P = 5.06^{-11}$), acetone ($r = 0.63$, $P = 1.64^{-6}$), lactate ($r = 0.64$, $P = 6.03^{-7}$), n-butyrate ($r = 0.82$, $P = 5.89^{-13}$), propionate ($r = 0.59$, $P = 8.26^{-6}$), and acetate ($r = 0.71$, $P = 1.23^{-8}$) concentrations in EBC, suggesting that these EBC metabolites might be suitable as inflammatory outcome measures.

Multidimensional integrated model for assessment of pharmacological treatment

In the 14 COPD study subjects, multidimensional pairwise discrimination models were built (Table 4). The multidimensional models discriminated between visit 1 versus visit 3 (accuracy = 71.5%, $P < 0.01$), mainly based on sputum supernatant PGE_2 and FEV_1/FVC ratio; between visit 1 versus visit 4 (accuracy = 82.5%, $P < 0.01$), mainly based on EBC formate, e-noses, and EBC PGE_2; between visit 3 versus visit 4 (accuracy = 74.6%, $P < 0.01$), mainly based on sputum PGE_2 and EBC acetate concentrations.

The multidimensional models showed higher accuracy than the models based on spirometry alone (Table 5). The sensitivity and specificity reported in Table 5 follow the standard definitions of proportion of true positives and proportion of true negatives, respectively. It is worth noting that

they appear equal for all the comparisons. Furthermore, score plots from PLS analysis are centrosymmetric. This seeming uncommon behaviour is fully understandable given the pairwise nature of the analyses performed. In a multilevel PLS, between subject variation is separated from within subject variation by subtracting the individual specific average. In the two class cases, this construction leads to a matrix with a two block structure with opposite signs, thus producing symmetric PLS scores. During cross-validation, when a multilevel PLS model is built from the training set, the entire variation splitting procedure is performed (van Velzen et al, 2008; Westerhuis et al, 2010). The procedure should, therefore, be adapted to keep the paired data structure both in the training and in the test set. As a result, complete individuals are left out of the training set (per individual validation, not per sample). At each step, if a sample of one individual is mistaken, inevitably the other is mistaken in the opposite way, leading to a fully symmetric confusion matrix. Of course, this symmetry is broken when considering more than just two time points.

DISCUSSION

Correlations

In the EBC, the strong correlations between PGE_2 and small molecular weight metabolites detected by NRM spectroscopy are unlikely to be explained by individual variability in aerosol particle formation (Effros et al, 2003) as there was no correlation EBC 15-F_{2t}-isoprostane and EBC metabolites nor between EBC PGE_2 and EBC 15-F_{2t}-isoprostane.

Persistent neutrophilic airway inflammation is a typical characteristic of COPD, which persists even after smoking cessation and sputum neutrophil cell counts are a direct measure of airway inflammation which can be used for assessing the anti-inflammatory effects of drugs (Simpson et al, 2014). We report a negative correlation between the response of some quartz crystal sensors, in terms of relative changes in sensor frequency $[(f\text{max}-f0)/f0]$ (Hz), and sputum neutrophil cell

counts. These data suggest that the quartz crystal sensor e-nose is potentially useful for assessing neutrophilic airway inflammation and that the higher neutrophilic airway inflammation the lower e-nose response.

The response of 15 out of 32 carbon polymer sensors was correlated with EBC phenol concentrations. However, the biological meaning of these data has to be defined. There was no correlation between any carbon polymer sensor and the inflammatory outcomes measured in this study, including sputum neutrophil and macrophages cell counts, PGE_2 concentrations in sputum supernatants and EBC, 15-F_{2t}-isoprostane concentrations in sputum supernatants and EBC. However, these findings do not exclude the potential utility of carbon polymer sensor e-nose for assessing airway inflammation as 1) inflammation that characterizes COPD is a complex, heterogeneous, pathophysiological process involving many mediators, whereas only a limited number of inflammatory outcomes was measured in the present study; 2) correlations were studied in 14 patients with COPD considering all 4 visits (n = 56) in a time frame of 10 weeks during which pharmacological treatment was changed three times: this might have caused different effects on e-nose sensors and inflammatory outcome measure, thus, resulting in non-significant correlations.

References

1. Alonso, A., Marsal, S., and Julià, A. (2015). Analytical methods in untargeted metabolomics: state of the art in 2015. *Front. Bioeng. Biotechnol.* 3, 23. doi: 10.3389/fbioe.2015.000232.

2. Bertini, I., Luchinat, C., Miniati, M., Monti, S., and Tenori, L. (2014). Phenotyping COPD by ^{1}H NMR metabolomics of exhaled breath condensate. *Metabolomics* 10, 302-311. doi:10.1007/s11306-013-0572-3

3. Bijlsma, S., Bobeldijk, I,, Verheij, E,R,, Ramaker, R., Kochhar, S., Macdonald, I.A., van Ommen, B., and Smilde, A.K. (2006). Large-scale human metabolomics studies: a strategy for data (pre-) processing and validation. *Anal. Chem.* 78, 567-574. doi: 10.1021/ac051495j

4. Bofan, M., Mores, N., Baron, M., Dabrowska, M., Valente, S., Schmid, M., Trové, A., Conforto, S., Zini, G., Cattani, P., Fuso, L., Mautone, A., Mondino, C., Pagliari, G., D'Alessio, T., and Montuschi, P. (2013) Within-day and between-day repeatability of measurements with an electronic nose in patients with COPD. *J. Breath Res.* 7(1), 017103. doi: 10.1088/1752-7155/7/1/017103

5. Boulesteix A-L. (2015) Package "WilcoxCV". Available at: https://cran.r-project.org/web/packages/WilcoxCV/WilcoxCV.pdf (accessed: 9 January 2018)

6. Cover, T., Hart, P. (1967). Nearest neighbor pattern classification. *IEEE Trans. Inf. Theory* 13, 21–27. doi: 10.1109/TIT.1967.1053964

7. De Laurentiis, G., Paris, D., Melck, D., Maniscalco, M., Marsico, S., Corso, G., Motta, A., and Sofia, M. (2008). Metabonomic analysis of exhaled breath condensate in adults by nuclear magnetic resonance spectroscopy. *Eur. Respir. J.* 32, 1175-1183. doi: 10.1183/09031936.00072408

8. Dieterle, F., Ross, A., Schlotterbeck, G., and Senn, H. (2006). Probabilistic quotient normalization as robust method to account for dilution of complex biological mixtures. Application in ^{1}H NMR metabonomics. *Anal. Chem.* 78, 4281–4290. doi: 10.1021/ac051632c

9. Djukanovic, R., Sterk, P.J., Fahy, J.V., and Hargreave, F.E. (2002). Standardised methodology of sputum induction and processing. *Eur. Respir. J.* 37(Suppl), 1s–2s. doi: 10.1183/09031936.02.00000102

10. Dragonieri, S., Schot, R., Mertens, B.J., Le Cessie, S., Gauw, S.A., Spanevello, A., Resta, O., Willard, N.P., Vink, T.J., Rabe, K.F., Bel, E.H., and Sterk, P.J. (2007). An electronic nose in the discrimination of patients with asthma and controls. *J. Allergy. Clin. Immunol.* 120, 856-862. doi: 10.1016/j.jaci.2007.05.043

11. Effros, R.M., Biller, J., Foss, B., Hoagland, K., Dunning, M.B., Castillo, D., Bosbous, M., Sun, F., and Shaker, R. (2003). A simple method for estimating respiratory solute dilution in exhaled breath condensates. *Am. J. Respir. Crit.Care Med.* 168,1500-1505. doi: 10.1164/rccm.200307-920OC

12. Efthimiadis, A., Spanevello, A., Hamid, Q., Kelly, M.M., Linden, M., Louis, R., Pizzichini, M.M., Pizzichini, E., Ronchi, C., Van Overvel, F., and Djukanović R. (2002). Methods of sputum processing for cell counts, immunocytochemistry and in situ hybridisation. *Eur. Respir. J.* 37 (Suppl), 19s-23s. doi: 10.1183/09031936.02.00001902

13. Fens, N., Zwinderman, A.H., van der Schee, M.P., de Nijs, S.B., Dijkers, E., Roldaan, A.C., Cheung, D., Bel, E.H., and Sterk, P. (2009). Exhaled breath profiling enables discrimination of chronic obstructive pulmonary disease and asthma. *Am. J. Respir. Crit. Care Med.* 180, 1076-1082. doi: 10.1164/rccm.200906-0939OC

14. Hahsler, M., Hornik, K., and Buchta, C. (2008). Getting things in order: an introduction to the R package seriation. *J. Stat. Softw.*. 25, 1-34. doi: 10.18637/jss.v025.i0315.

15. Ihaka, R., and Gentleman, R. (1996). R: A Language for Data Analysis and Graphics. *J. Comput. Graph. Stat.* .5, 299–314. doi: 10.1080/10618600.1996.10474713

16. Lewis, N.S. (2004). Comparisons between mammalian and artificial olfaction based on arrays of carbon black-polymer composite vapor detectors. *Acc. Chem. Res.* 37, 663-672. doi: 10.1021/ar030120m

17. Montuschi, P., Collins, J.V., Ciabattoni, G., Lazzeri, N., Corradi, M., Kharitonov, S.A., and Barnes, P.J. (2000). Exhaled 8-isoprostane as an in vivo biomarker of lung oxidative stress in patients with COPD and healthy smokers. *Am. J. Respir. Crit. Care Med.* 162, 1175-1177. doi: 10.1164/ajrccm.162.3.2001063

18. Montuschi, P., Paris, D., Melck, D., Lucidi, V., Ciabattoni, G., Raia, V., Calabrese, C., Bush, A., Barnes, P.J., and Motta, A. (2012). NMR spectroscopy metabolomic profiling of exhaled

breath condensate in patients with stable and unstable cystic fibrosis. *Thorax* 67, 222-228. doi: 10.1136/thoraxjnl-2011-200072

19. Montuschi, P., Ragazzoni, E., Valente, S., Corbo, G., Mondino, C., Ciappi, G., and Ciabattoni, G. (2003). Validation of 8-isoprostane and prostaglandin E_2 measurements in exhaled breath condensate. *Inflamm. Res.* 52, 502-507. doi: 10.1007/s00011-003-1212-6

20. Montuschi, P., Santonico, M., Mondino, C., Pennazza, G., Mantini, G., Martinelli, E., Capuano, R., Ciabattoni, G., Paolesse, R., Di Natale, C., Barnes, P.J., and D'Amico, A. (2010). Diagnostic performance of an electronic nose, fractional exhaled nitric oxide, and lung function testing in asthma. *Chest* 137, 790-796. doi: 10.1378/chest.09-1836

21. Motta, A., Paris, D., Melck, D., De Laurentiis, G., Maniscalco, M., Sofia, M., and Montuschi, P. (2012). Nuclear magnetic resonance-based metabolomics of exhaled breath condensate: methodological aspects. *Eur. Respir. J.* 39, 498-500. doi: 10.1183/09031936.00036411

22. Paggiaro, P., Chanez, P., Holz, O., Ind, P.W., Djukanovic, R., Maestrelli, P., and Sterk, P.J. (2002). Sputum induction. *Eur. Respir. J.* 37(Suppl), 3s–8s. doi: 10.1183/09031936.02.00000302

23. Revelle, W. (2017). An overview of the psych package. Package “psych” (version 1.6.9). Available at: https://cran.r-project.org/web/packages/psych/vignettes/overview.pdf. (accessed: 9 January 2018).

24. Röck, F., Barsan, N., and Weimar, U. (2008). Electronic nose: current status and future trends. *Chem. Rev.* 108, 705-711. doi: 10.1021/cr068121q

25. Simpson, J.L., Powell, H., Baines, K.J., Milne, D., Coxson, H.O., Hansbro, P.M., and Gibson, P.G. (2014). The effect of azithromycin in adults with stable neutrophilic COPD: a double blind randomised, placebo controlled trial. *PLoS One* 9(8), e105609. doi: 10.1371/journal.pone.0105609

26. van Velzen, E.J., Westerhuis, J.A., van Duynhoven, J.P., van Dorsten, F.A., Hoefsloot, H.C., Jacobs, D.M., Smit, S., Draijer, R., Kroner, C.I., and Smilde, A.K. (2008). Multilevel data analysis

of a crossover designed human nutritional intervention study. *J. Proteome Res.* 7, 4483–4491. doi: 10.1021/pr800145

28. Wang, Z., Ciabattoni, G., Créminon, C., Lawson, J., Fitzgerald, G.A., Patrono, C., and Maclouf, G. (1995). Immunological characterization of urinary 8-epi-prostaglandin $F_{2\alpha}$ excretion in man. *J. Pharmacol. Exp. Ther.* 275, 94-100.

29. Warnes, G.R., Bolker, B., Bonebakker, L., Gentleman, R., Liaw, W.H.A., Lumley, T., Maechler, M., Magnusson, A., Moeller, S., Schwartz, M., and Venables, B. (2016). Various R programming tools for plotting data. Package "gplots" (version 3.0.1). Available at: https://cran.r-project.org/web/packages/gplots/gplots.pdf. (accessed: 9 January 2018)

30. Westerhuis, J.A., van Velzen, E.J., Hoefsloot, H.C., and Smilde, A.K. (2010). Multivariate paired data analysis: multilevel PLSDA versus OPLSDA. *Metabolomics* 6, 119–128.

31. Wilcoxon, F. (1945). Individual comparisons by ranking methods. *Biom. Bull.* 1, 80-83

32. Wilson, A.D., and Baietto, M. (2009). Applications and advances in electronic-nose technologies. *Sensors (Basel)* 9, 5099-5148. doi: 10.3390/s90705099

33. Wishart, D.S., Tzur, D., Knox, C., Eisner, R., Guo, A.C., Young, N., Cheng, D., Jewell, K., Arndt, D., Sawhney, S., Fung, C., Nikolai, L., Lewis, M., Coutouly, M.A., Forsythe, I., Tang, P., Shrivastava, S., Jeroncic, K., Stothard, P., Amegbey, G., Block, D., Hau, D.D., Wagner, J., Miniaci, J., Clements, M., Gebremedhin, M., Guo, N., Zhang, Y., Duggan, G.E., Macinnis, G.D., Weljie, A.M., Dowlatabadi, R., Bamforth, F., Clive, D., Greiner, R., Li, L., Marrie, T,, Sykes, B.D., Vogel, H,J,, and Querengesser, L.. HMDB: the Human Metabolome Database. (2007). *Nucleic Acids Res.* 35, D521-526. doi: 10.1093/nar/gkl923

Figure legends

s-Figure 1. Nuclear Overhauser effect spectroscopy (NOESY) ^{1}H-NMR spectra of exhaled breath condensate samples obtained from one randomly selected COPD study subject at visit 1 (red), visit 2 (green), visit 3 (light blue), and visit 4 (purple).

s-Figure 2. Pairwise partial least square (PLS) analysis of exhaled breath condensate in 14 subjects with COPD at visit 1 (red dots) and visit 4 (blue dots) (classification accuracy = 0.72, P = 0.01).

s-Figure 3. A) Formate peak in exhaled breath condensate (EBC) NMR spectra obtained from 14 patients with COPD at visit 1 (red lines) and visit 4 (green lines). B) Box-and-Whiskers plot of formate levels in EBC obtained by analysing NMR spectra from 14 patients with COPD at visit 1 and visit 4. Median arbitrary units and median absolute deviation are shown.

s-**Table 1.** Classification accuracies and P values among different pharmacological treatments from visit 1 to visit 4 based on a monodimensional PLS model built on EBC NMR spectroscopy data in 14 patients with COPD. The significant variables in the univariate analysis are also reported (Wilcoxon signed-rank test P values).

s-

**Comparison**	**Overall accuracy**	**Variable (P value)**
Visit 1 vs. Visit 2	42.0% (P > 0.05)	-
Visit 1 vs. Visit 3	55.3% (P > 0.05)	-
Visit 1 vs. Visit 4	72.0% (0.01)	Formate (0.029)
Visit 2 vs. Visit 3	53.3% (P > 0.05)	-
Visit 2 vs. Visit 4	63.3% (P > 0.05)	-
Visit 3 vs. Visit 4	57.3% (P > 0.05)	Acetate (0.009)

Abbreviations: EBC, exhaled breath condensate; NMR, nuclear magnetic resonance; PLS, partial least squares.

s-Table 2. F_ENO values in 14 patients with COPD at visit 1 to visit 4.*

	V1 (n = 14)	V2 (n = 14)	V3 (n = 14)	V4 (n = 14)	Overall P value
F_ENO (ppb)	21.9 (12.7-28)	17.6 (12.3-20.3)	18.5 (11.8-29)	13.2 (11.8-21.7)	0.35

*Data are expressed as medians and interquartile ranges. Within-group comparisons were performed with Friedman test and Wilcoxon matched-pairs signed rank test. Abbreviations: F_ENO, fraction of exhaled nitric oxide; V, visit.

s-Table 3. PGE_2 and 15-F_{2t}-isoprostane concentrations in sputum and EBC*.

	V1	V2	V3	V4	Overall P value
n	13	13	13	13	
Sputum PGE_2 (pg/ml)	18.6 (2-24.6)[1]	3.8 (2-54.9)	40.1 (8.3-47)[1,2]	10.4 (3.5-20.8)[2]	0.04
	V1	V2	V3	V4	Overall P value
n	9	9	9	9	
Sputum 15-F_{2t}-isoprostane (pg/ml)	25 (9.9-45.5)	25.5 (13.2-45.4)	33.2 (7.7-41.5)	13.5 (7.8-26.2)	0.84
	V1	V2	V3	V4	Overall P value
n	12	12	12	12	
EBC PGE_2 (pg/ml)	18.7 (10.6-30.4)	19.1 (11.9-55.1)	14.7 (7-26.1)	11.3 (4-18.7)	0.19
	V1	V2	V3	V4	Overall P value
n	14	14	14	14	
EBC 15-F_{2t}-isoprostane (pg/ml)	18.3 (1.4-37)	24 (9.7-35.7)	20.8 (15.1-32.8)	21.3 (8.1-32.7)	0.60

*Data are expressed as median and interquartile range. Within-group, between-visit comparisons were performed with Friedman's test. If overall P was lower than 0.05, considered significant, Wilcoxon matched-pairs signed rank test was performed.
[1]: P = 0.021; [2]: P = 0.008.
One patient with COPD had undetectable sputum PGE_2 concentrations at all visits (4 samples). Five patients with COPD had undetectable sputum 15-F_{2t}-isoprostane concentrations at all visits (20 samples). Two patients with COPD had undetectable EBC PGE_2 concentrations at all visits (8 samples). At undetectable samples, an arbitrary concentration value of 1 pg/ml, corresponding to 50% of the analytical technique detection limit (2 pg/ml), was assigned.
Abbreviations: EBC, exhaled breath condensate; PGE_2, prostaglandin E_2; V, visit.

s-Table 4. Within-group comparison of percentage sputum cell counts in the 8 patients with COPD who had a complete set of sputum slides (visit 1 to visit 4)*.

	V1	V2	V3	V4	P value
n	8	8	8	8	
Neutrophils, %	82.5 (57.3-91.6)	85.5 (77.6-95.5)	86.5 (72.3-93.9)	83.3 (68.4-89.6)	0.55
Macrophages, %	10.5 (4.1-39.8)	11.3 (2.8-17.4)	10 (1.7-15)	7.8 (4.4-18)	0.91
Eosinophils, %	0 (0-0.5)	0 (0-0.8)	0.5 (0-2.9)	0.5 (0-3.3)	0.20
Lymphocytes, %	0 (0-0.4)	0 (0-0.5)	0 (0-0.4)	0 (0-0.4)	0.87

*Data are expressed as medians and interquartile range. Percentage of basophil and bronchial epithelial cell counts was 0 and is not shown. Within-group comparisons were performed with Friedman test. Abbreviation: V, visit.

s-Table 5. Percentage sputum cell counts in all valid sputum slides (n = 43)*.

	V1	V2	V3	V4	P value
n	10	12	11	10	
Neutrophils, %	85.8 (67.8-93.4)	87.5 (78-96)	87 (73-89)	83.5 (72.1-90.8)	n.s.
Macrophages, %	10.5 (2.9-25.3)	7.8 (1.8-17.4)	11 (3-13.5)	7.8 (4-18)	n.s.
Eosinophils, %	0.3 (0-0.6)	0 (0-1)	0 (0-1)	0.5 (0-2.8)	n.s.
Lymphocytes, %	0 (0-0.1)	0 (0-0.4)	0 (0-0)	0 (0-1)	n.s.

*Data are expressed as medians and interquartile range. Thirteen sputum slides were excluded due to squamous cell counts > 30%. Sputum cell counts are expressed as a percentage of total non-squamous cells. Percentage of basophils and bronchial epithelial cells was 0. Comparisons were performed with Mann-Withney test. Abbreviations: n.s., not significant for all comparisons within the group; V, visit.
