# Supplementary material for: Breathomics for Assessing the Effects of Treatment and Withdrawal With Inhaled Beclomethasone/Formoterol in Patients With COPD
Source: Front Pharmacol. 2018 Apr 17;9:258. doi: 10.3389/fphar.2018.00258 (PMC5914154; doi:10.3389/fphar.2018.00258)
Supplement: Supplementary file 7 [file Presentation2.pdf]

Figure legends

s-Figure 1. Nuclear Overhauser effect spectroscopy (NOESY) ^{1}H-NMR spectra of exhaled breath condensate samples obtained from one randomly selected COPD study subject at visit 1 (red), visit 2 (green), visit 3 (light blue), and visit 4 (purple).

s-Figure 2. Pairwise partial least square (PLS) analysis of exhaled breath condensate in 14 subjects with COPD at visit 1 (red dots) and visit 4 (blue dots) (classification accuracy = 0.72, P = 0.01).

s-Figure 3. A) Formate peak in exhaled breath condensate (EBC) NMR spectra obtained from 14 patients with COPD at visit 1 (red lines) and visit 4 (green lines). B) Box-and-Whiskers plot of formate levels in EBC obtained by analysing NMR spectra from 14 patients with COPD at visit 1 and visit 4. Median arbitrary units and median absolute deviation are shown.
