# Supplementary figures and images for: Breathomics for Assessing the Effects of Treatment and Withdrawal With Inhaled Beclomethasone/Formoterol in Patients With COPD
Source: Front Pharmacol. 2018 Apr 17;9:258. doi: 10.3389/fphar.2018.00258 (PMC5914154; doi:10.3389/fphar.2018.00258)

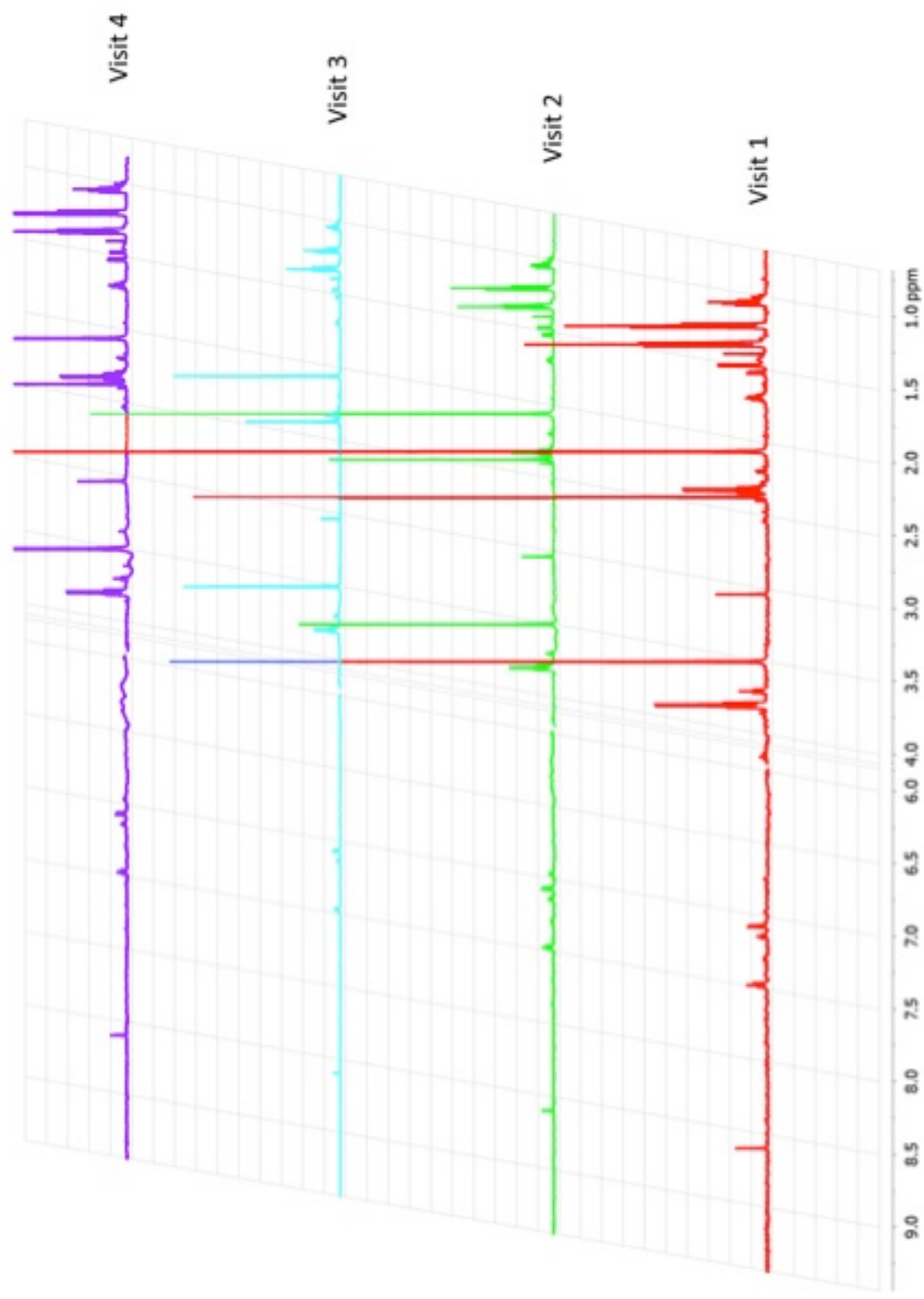

Supplement: Supplementary file 8 [file Image1.pdf]

Accuracy: 72.0%

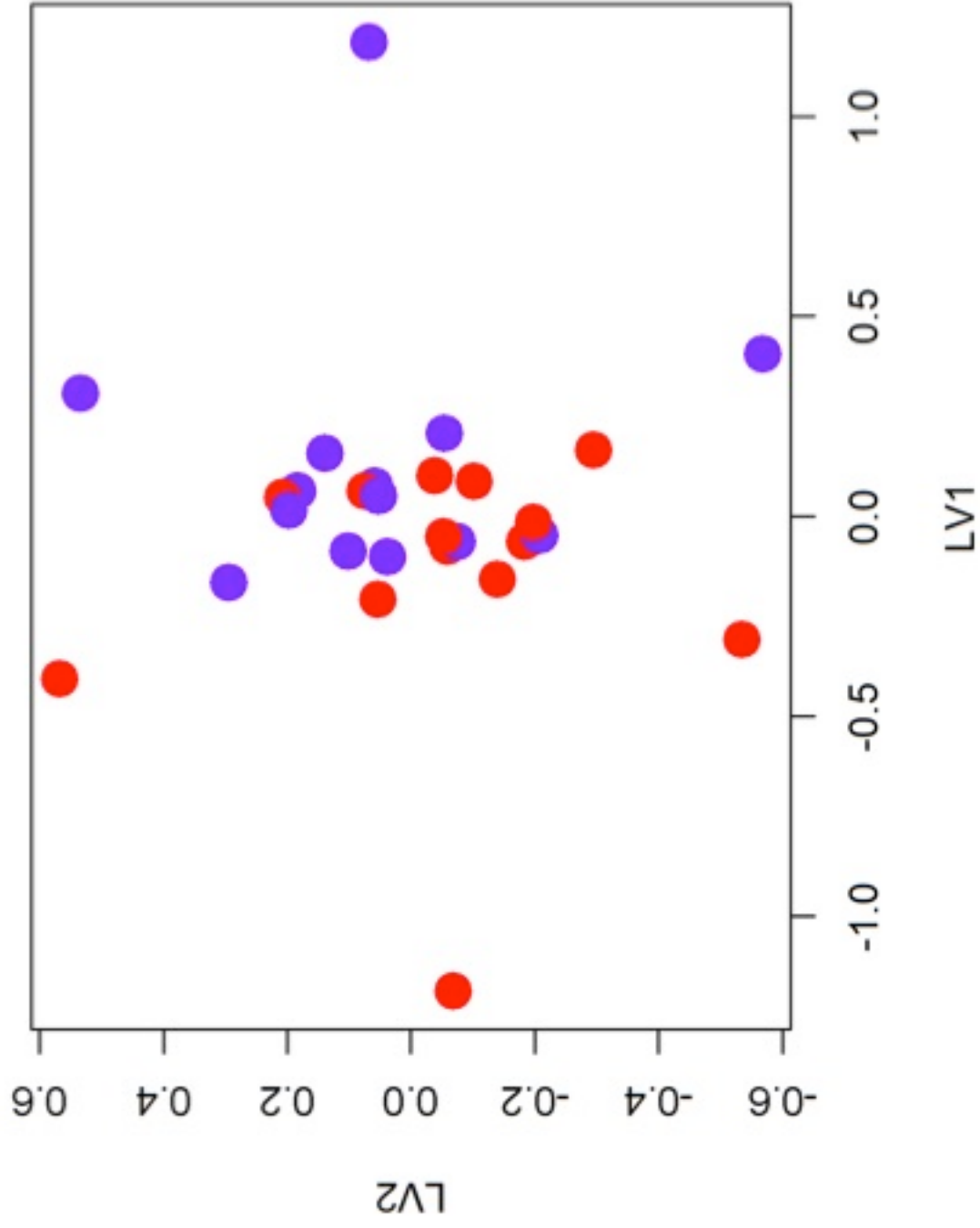

Supplement: Supplementary file 9 [file Image2.pdf]

A)

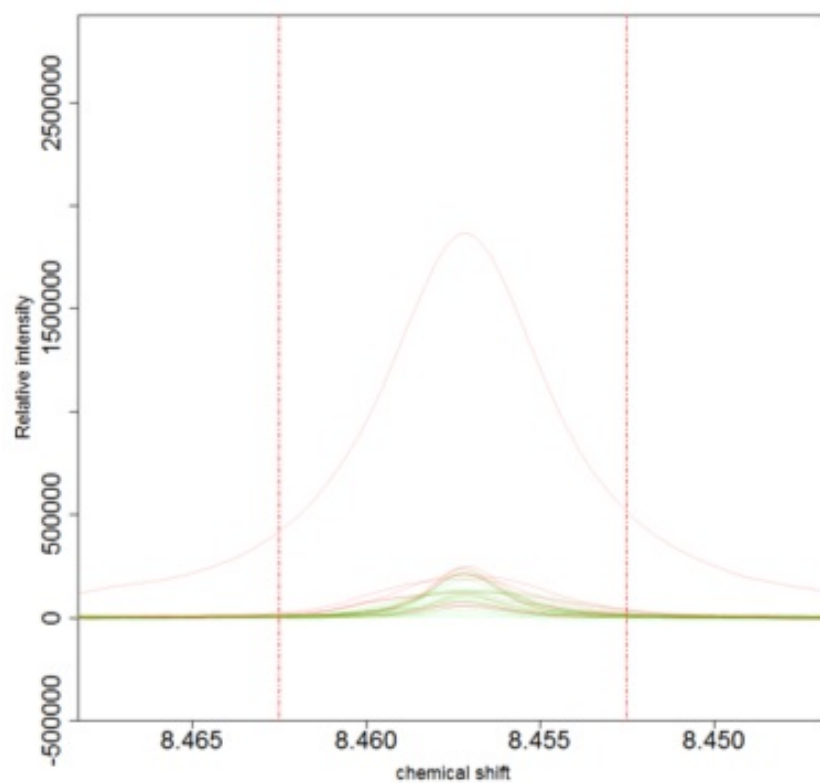

B)

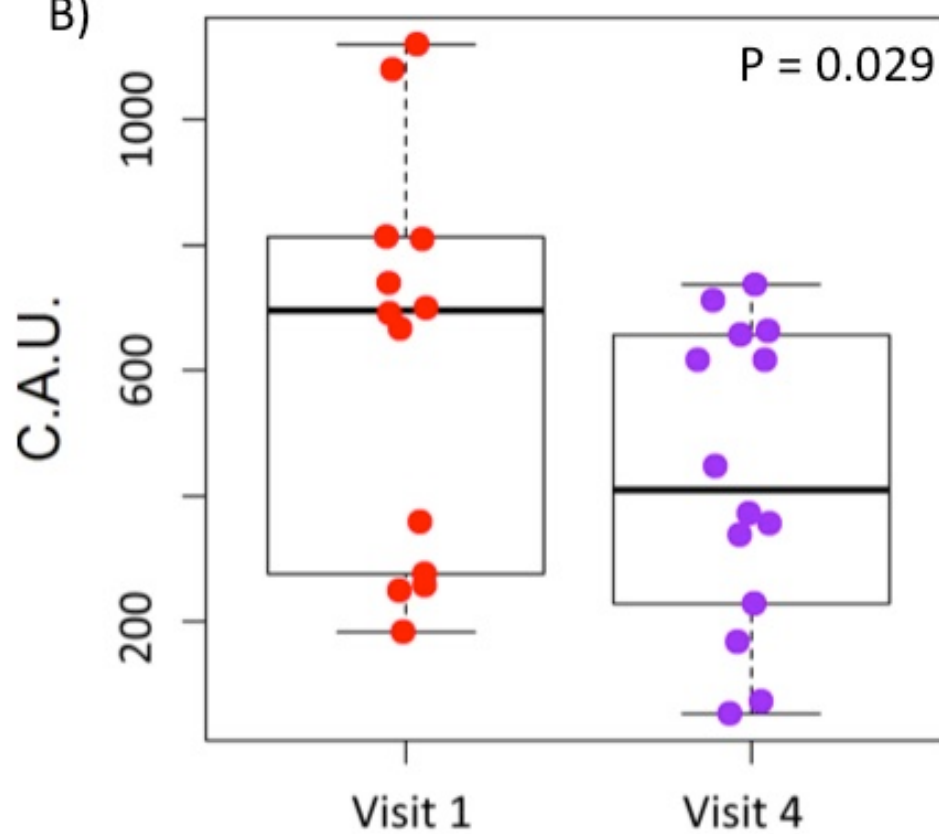

Supplement: Supplementary file 10 [file Image3.pdf]
